# Structure–Property–Degradation Relationships in PHBV Biocomposites Filled with Cellulose, Walnut Shell, and Wood Fibres

**DOI:** 10.3390/ma19214638

**Published:** 2026-10-31

**Authors:** Patrycja Szydłowska, Patrycja Bazan, Stanisław Kuciel

**Affiliations:** 1 Faculty of Materials Engineering and Physics, PK Krakow University of Technology, Jana Pawła II 37, 31-864 Kraków, Poland; patrycja.szydlowska@student.pk.edu.pl (P.S.); patrycja.bazan@pk.edu.pl (P.B.); 2 Interdisciplinary Center for Circular Economy, Cracow University of Technology, Warszawska 24, 31-155 Kraków, Poland

**Keywords:** lignocellulosic reinforcement, interfacial morphology, surface wettability, water sorption, mechanical performance, environmental ageing

## Abstract

PHBV composites incorporating 10 and 20% by weight of cellulose, walnut shell particles, or wood filler were produced under consistent extrusion and injection-moulding conditions for comparative analysis. The study assessed morphology, wettability, water absorption, mechanical properties at 23 and 80 °C, thermal decomposition, and alterations in mechanical properties following exposure to a detergent solution at 60 °C. Among the mean values reported, PHBV 20W exhibited the highest modulus at 23 °C (7403 MPa), while PHBV 10C demonstrated the highest impact strength (8.9 kJ/m^2^). At a filler content of 20% by weight, the tensile strength was observed to be lower than that of the matrix. After 35 days, water absorption varied from 1.3% for PHBV to 14.7% for PHBV 20W. Thermogravimetric analysis (TGA) results revealed that the direction of the shift in the temperature at 5% mass loss was filler-dependent, with values ranging from 229.2 to 272.2 °C, compared to 267.2 °C for PHBV. Exposure to detergent resulted in reductions in strength and elongation, with PHBV 20R and 20W experiencing a marked decrease in modulus.

## 1. Introduction

Polyhydroxyalkanoates (PHAs) constitute a class of bio-based polyesters synthesised by microorganisms, notable for their ability to biodegrade across diverse environments. Among these, poly(3-hydroxybutyrate-co-3-hydroxyvalerate) (PHBV) stands out due to its biodegradability and compatibility with conventional thermoplastic processing techniques. Although PHBV offers advantages over the PHB homopolymer, it is constrained by a relatively limited processing window, high rigidity, low elongation at break, and a tendency towards brittle fracture. These constraints hinder its application in thin-walled components and injection-moulded products, which demand concurrent strength, damage resistance, and stability of properties during use. Blending PHBV with more ductile polymers may enhance its crack resistance; however, this approach results in multiphase systems whose properties are contingent upon the morphology and compatibility of the components [1,2]. An alternative modification strategy involves incorporating natural-origin fillers, which can decrease the proportion of the costly polymer matrix, tailor the material’s functional properties, and maintain its potential for biological management post-use [3].

Lignocellulosic fillers are appealing due to their renewable origin, low density, availability, and capacity to manage by-products from the wood and agri-food industries. Nevertheless, their incorporation into PHBV does not consistently result in enhanced strength. The mechanical properties of a composite are influenced by factors such as the type of filler, particle size and shape, fibre form factor, mass fraction, surface chemistry, degree of dispersion, and the quality of the interfacial connection between phases. Effective adhesion facilitates the transfer of stresses from the matrix to the more rigid natural phase. Conversely, inadequate wetting, interfacial voids, and agglomerates act as local stress concentrators and sites for crack initiation [3,4,5]. Consequently, increasing the filler content may enhance the modulus of elasticity but simultaneously diminish strength and formability, particularly when the matrix is insufficient to fully wet the particle surface or when intensified filler-filler contacts occur [4].

Natural lignocellulosic fillers exhibit variation not only in morphology and chemical composition but also in origin, availability, and industrial application. Cellulose fibres, derived from lignocellulosic feedstocks, are commercially accessible in a wide array of dimensions and purities, serving as reinforcing and functional fillers in polymeric and other technical materials [5,6]. Their low density, renewable nature, stiffness, and surface functionality have heightened interest in their application within bio-based polymer composites [5]. Similarly, wood fibres, sourced from lignocellulosic resources, are extensively utilized as reinforcing elements in wood–polymer composites and other technical materials, where their elongated morphology and high aspect ratio enhance stiffness and stress transfer [5,7]. The regional availability of these fibres is particularly significant in countries with a developed forestry sector. For instance, in Poland, forests spanned approximately 9.29 million hectares in 2024, accounting for 29.6% of the national territory, while annual wood removals surpassed 41 million m^3^, underscoring the substantial regional availability of wood-derived raw materials and related processing streams [8].

Walnut shells constitute a distinct category of lignocellulosic resources, as they are produced as an agro-industrial by-product during the nut processing phase, following the extraction of the edible kernel [9,10]. The proportion of the shell in relation to the entire walnut mass varies based on the cultivar and processing conditions, typically ranging from 40% to 60% by weight [9]. Given the substantial global production of walnuts, this results in significant quantities of lignocellulosic residue that necessitate management or valorisation. Manterola-Barroso et al. have estimated that with global walnut production surpassing 4.5 million tonnes, approximately 2.2 million tonnes of walnut shell by-product could be generated [9]. Currently, walnut shells are utilized or explored for applications such as abrasives, adsorption and filtration media, precursors for activated carbon, and as particulate fillers in polymeric and construction materials [9,10]. Their hardness, lignocellulosic composition, relatively high lignin content, renewable nature, and availability have thus spurred interest in their valorisation within circular-material strategies [9].

The variations in origin, availability, morphology, and industrial status of cellulose, walnut shell particles, and wood fibres justify their comparison within the same PHBV matrix. The distinct geometries, aspect ratios, and surface characteristics of these particles and fibres are anticipated to affect dispersion, filler–matrix interactions, stress transfer, and moisture sensitivity in diverse manners [3,7,11,12]. By conducting this comparison at identical filler contents and under uniform extrusion and injection-moulding conditions, the impact of technological variables is minimized, allowing for a more direct evaluation of the lignocellulosic phase’s role in determining composite performance.

Cellulose, walnut shell particles, and wood fibres represent three distinct types of natural phase morphology. Fine or short-fibrous cellulose offers a relatively high interfacial area with the polymer matrix. When adequately dispersed and well bonded to PHBV, it can facilitate stress transfer and modify PHBV crystallization through heterogeneous nucleation. However, the extent of these effects is significantly influenced by the dimensions, loading, dispersion, and interfacial compatibility of the filler [13,14,15].

However, its high surface energy may lead to agglomerate formation if mixing conditions do not facilitate effective particle separation. Walnut shell particles, characterised by their irregular shape and developed surface topography, can enhance mechanical anchoring within the matrix. Nonetheless, they may also result in debonding, pull-out, and local stress concentration due to inadequate adhesion. Conversely, wood fibres can establish more efficient load transfer paths and significantly increase the modulus of elasticity owing to their higher form factor. However, their effectiveness is contingent upon orientation, changes in fibre dimensions during extrusion and injection moulding, and the quality of the phase boundary [3,4,7,11,14]. Research on PHBV composites suggests that modifying filler surfaces or employing reactive compatibilisation can enhance dispersion and adhesion. Nevertheless, optimal conditions must be tailored to the specific chemistry and morphology of each filler [11,14].

A significant limitation of composites containing a lignocellulosic phase is their hydrophilic nature. The hydroxyl groups present increase water affinity, leading to moisture absorption that can cause filler swelling, dimensional alterations, weakening of the bond with the matrix, and the formation of microcracks and additional pathways for water transport. These phenomena impact the retention of mechanical properties during hydrothermal ageing and environmental exposure [11,12,16]. Concurrently, increased water availability and an expanded interfacial surface may promote microbial colonisation and expedite the degradation of the PHA matrix. Nevertheless, this relationship is not straightforward, as the kinetics of transformations are also affected by factors such as porosity, particle size, degree of dispersion, and the content of cellulose, hemicellulose, and lignin. Lignin, in particular, can restrict enzyme access and slow the degradation of the lignocellulose fraction under certain conditions [1,17,18]. Consequently, the evaluation of PHBV composites should be based on a comprehensive analysis of surface wettability, water absorption, phase boundary morphology, and changes in mechanical properties, rather than solely on the initial modulus or strength value.

PHBV composites incorporating nanocellulose, walnut shell meal, wood, hemp, and linen fibres, along with other naturally derived fillers, have been extensively documented in the literature. These studies consistently demonstrate that the geometry of the dispersed phase and the quality of homogenisation significantly influence the processing, filler orientation, mechanical properties, and water resistance of the composites [3,4,7,12]. Nevertheless, direct comparison of data is complicated by variations in the grade and content of 3-hydroxyvalerate in PHBV, filler contribution, particle size distribution, surface treatment, drying, extrusion and forming parameters, as well as conditioning and degradation conditions. There remains a paucity of studies that simultaneously compare cellulose, walnut shell particles, and wood fibres within a single controlled system, maintaining consistent contents and identical processing conditions, while also analysing microstructure, surface properties, water absorption, mechanical behaviour, and degradation changes. Such an experimental framework is essential to minimise the impact of technological variables and to compare the performance of the formulations under common processing settings, and the role within the composite [3,4,7,12,18].

Mazur et al. [19] conducted an investigation into PHBV composites incorporating cellulose, walnut shell, and wood fillers, specifically focusing on formulations with 7.5% by weight filler, and analyzed alterations in mechanical properties following environmental exposure. The current study extends this research by evaluating formulations with 10% and 20% by weight filler. This evaluation employs a comprehensive analysis of morphology, wettability, water absorption, mechanical properties at 23 °C and 80 °C, thermogravimetric analysis, and the retention of mechanical properties during continuous detergent exposure at 60 °C. The study’s contribution lies in the formulation-specific comparison; however, the use of these filler classes and the examination of aging are not presented as novel aspects.

## 2. Materials and Methods

### 2.1. Materials

ENMAT Y1000P is a thermoplastic polyhydroxyalkanoate (PHBV) engineered for processing through various techniques, such as injection moulding, thermoforming, blow moulding, and extrusion. ENMAT is produced by Tian An Biopolymer and distributed by Helian Polymers (Belfeld, Germany). The material is fully biodegradable and bio-based, making it appropriate for single-use products and bio-based composite applications. According to the manufacturer, the 3HV content in ENMAT Y1000P is approximately 1–2% by weight. or about 1 mol% [20]. The fundamental physical and mechanical properties, as specified by the manufacturer, are detailed in Table 1 [21].

Three plant-based fillers were employed to fabricate composites within the PHBV matrix: Arbocel^®^ BC 1000 technical cellulose (Kremer Pigmente, Aichstetten, Germany), REHOFIX^®^ 200 walnut meal (Rettenmaier, Rosenberg, Germany), and Lignocell^®^ CB 120 wood fibres (Rettenmaier, Rosenberg, Germany). These fillers varied in morphological structure, particle size, and surface characteristics, enabling an evaluation of the impact of filler type on the physical and mechanical properties, as well as changes in properties following degradation in an aqueous detergent solution at elevated temperatures of PHBV-based composites. The selection of fillers was guided by their derivation from renewable raw materials, market availability, and their potential to modify the mechanical and environmental properties of composite materials, while retaining a biodegradable PHBV matrix and incorporating renewable lignocellulosic constituents.

**Figure 1:**
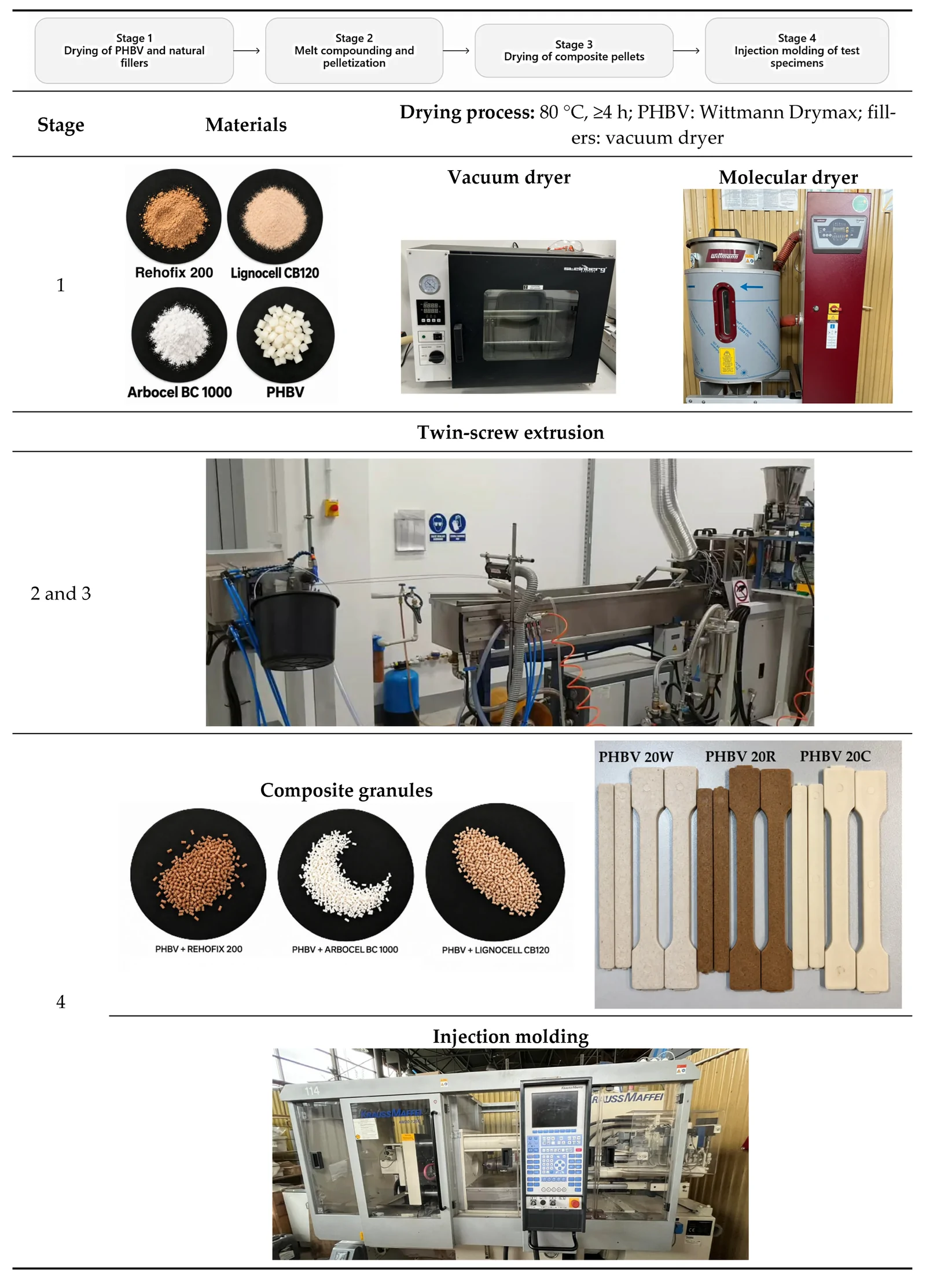
Illustration of the preparation process for PHBV and PHBV composites incorporating 10 and 20% by weight cellulose, walnut shell particles, or wood fibres. Stage 1—raw material preparation and drying; stage 2—extrusion and pelletisation; stage 3—pellet drying; stage 4—injection moulding of specimens.

The granulation of the tested thermoplastic composites was conducted using a laboratory extrusion line from Steer (Bangalore, India), which is equipped with an Omega 20H co-rotating twin-screw extruder (D = 20 mm, L/D = 44), two volumetric feeders, a cooling bath, and a granulator (STEER Engineering, Bengaluru, India). During the production of PHBV ENMAT Y1000P granulate, a temperature profile was used in accordance with the manufacturer’s recommendations for processing the material in the molten state. The temperature was gradually increased in the successive zones of the plasticizing system: from 150 °C in the supply zone, through 160 °C in the compression zone and 165 °C in the dosing zone, to 170 °C in the adapter and head area. The extruder screw operated at a speed of 60 rpm, with a total feed rate of 4 kg/h. The melt residence time was not quantified. Such a profile allows for gradual plasticization of the polymer and obtaining a homogeneous melt, while reducing the risk of excessive heat load on PHBV. This is particularly important due to the relatively narrow processing window of this polymer and its susceptibility to thermal degradation during prolonged exposure to elevated temperatures. The 150/160/165/170/170 °C profile used was therefore a compromise between ensuring proper plasticization of the material and minimizing undesirable changes in its structure during the granule production process. Standard ISO 3167 [22] multipurpose specimens were subsequently produced through injection moulding using a KM 40–125 Winner Krauss Maffei injection moulding machine (Munich, Germany). The injection parameters for PHBV included moulding zones set at 140 °C (I), 160 °C (II), 165 °C(III), and 170 °C (IV), a rotational speed of 50 rpm, and an injection pressure of 700 bar, with a mould temperature of 50 °C. Before extrusion and subsequent injection moulding, all additives and polymer granules were dried at 80 °C for at least four hours, following the manufacturers’ recommendations. The polymer granules were dried using a Wittmann Drymax Basic series molecular dryer (Wittmann, Austria). The fillers were dried in a Goldbrunn 450 laboratory vacuum oven (Expondo GmbH, Berlin, Germany). Following preliminary drying, the moisture content of the components was measured. The moisture content of the natural filler particles was determined using a moisture analyzer (model HX204, Mettler-Toledo AG, Greifensee, Switzerland) by measuring mass loss during drying at 80 °C until a target endpoint criterion (below 0.5%) was reached. Results were expressed as a percentage of the initial sample mass. The water content of the PHBV granules and composites was determined via coulometric Karl Fischer titration using a 917 coulometer equipped with an 874 Oven Sample Processor (Metrohm AG, Herisau, Switzerland). Figure 1 illustrates the technological process. Table 2 details the material designation, filler characteristics, and composite density.

### 2.2. Methods

The density of the composites was assessed using the hydrostatic method with a RADWAG WAS 22W analytical balance (Radwag, Radom, Poland), following the ISO 1183 standard [23]. Ethanol served as the reference liquid. Density was measured on samples after injection molding. The microstructure of the composites was examined after tensile testing via scanning electron microscopy (SEM; JEOL JSM-IT200, Tokyo, Japan). This analysis was conducted under low vacuum conditions at an acceleration voltage of 10 kV. Prior to imaging, samples were coated with a thin gold layer using the JEOL DII-29030SCTR sputter coater (Tokyo, Japan). The analysis of organic filler particles and their distribution within the polymer matrix was conducted using a Keyence VHX-5000 optical microscope (Osaka, Japan), equipped with a digital image capture and analysis system. Observations were conducted at magnifications of 50×, 100×, and 200× to evaluate the morphology, shape, orientation, and particle size distribution within the composites. Representative optical micrographs of the three fillers underwent a semi-quantitative image-based dimensional analysis. Image calibration utilized the scale bars embedded in the original micrographs. Due to the varying morphologies of the fillers, distinct two-dimensional descriptors were employed: apparent contrast-domain diameter for cellulose, projected major-axis length for walnut shell particles, and projected fibre length and width for wood fibres. The analysis aimed to provide comparative apparent dimensions of the fillers rather than a standardized particle-size distribution. Optical microscopy observations complemented SEM by offering a rapid and non-destructive evaluation of filler dispersion homogeneity on a larger observational scale, facilitating the detection of agglomerates and structural discontinuities. The micrographs depict specific local characteristics of the imaged specimens. They serve for qualitative analysis and do not measure interfacial defect density or determine statistically significant differences among formulations. The fracture surfaces of all five tested specimens from each material series were analyzed using SEM at various magnifications. A minimum of 10 micrographs were obtained. Images deemed representative of the morphology observed in the specimens were chosen for presentation based on qualitative visual evaluation.

The particle size distribution was assessed using an Anton Paar PSA 1190LD analyser (Anton Paar GmbH, Graz, Austria). In the volume-based distribution, D10, D50, and D90 represent the diameters below which 10%, 50%, and 90% of the particle volume are contained, respectively; D50 serves as the median of the distribution. The distribution width is characterized by the span parameter, as defined in Equation (1). It is important to note that equivalent diameters determined by laser diffraction do not correspond to fibre lengths or widths measured in two-dimensional images. The particle-size parameters are reported as: mean value from 5 measurements within a single test; SD (±) from 5 measurements on the same filler independently prepared aliquots. Particle-size distributions were determined using wet laser diffraction. The fillers were dispersed in water using ultrasound. The measurement duration was 1 min at maximum speed. Particle-size distributions were calculated using the Fraunhofer optical model. D10, D50, D90 and span are reported in Table 3.

(1) $$Span=\frac{\left(D90-D10\right)}{D50}$$

Static contact angles were measured at ambient temperature utilizing an optical goniometer (Advex Instruments, Brno, Czech Republic). Distilled water and diiodomethane served as probe liquids. Droplets, each with a volume of 5 µL, were deposited on the smooth surfaces of the specimens, and the contact angle was recorded 5 s post-deposition. For each formulation and probe liquid, five independent specimens were evaluated, with each specimen comprising five droplets. The droplets were considered as measurements within each specimen. The reported variation corresponds to the standard deviation between specimens. The dispersive and polar components of the surface free energy were calculated using the Owens–Wendt–Rabel–Kaelble (OWRK) method. Surface tension values of 21.8 mN/m and 51.0 mN/m were used for the dispersive and polar components of water, respectively, while values of 50.8 mN/m and 0 mN/m were used for diiodomethane. The total surface free energy was determined as the sum of both components. Results are presented as mean ± standard deviation.

Basic equation of OWRK:

(2) $$\gamma_{L}(1+cos\;\theta )=2(\sqrt{\gamma_{S}^{d}\gamma_{L}^{d}}+\sqrt{\gamma_{S}^{p}\gamma_{L}^{p}})$$

For diiodomethane, the zero polar component is assumed; therefore, the dispersion component of the solid surface can be determined directly:

(3) $$\gamma_{S}^{d}\;=\;\gamma_{DIM}\;{\left(\frac{1\;+\;cos\;\theta_{DIM}}{2}\right)}^{2}$$

Then, the polar component is determined from the measurement for water:

(4) $$\gamma_{S}^{p}\;=\;{\left[\frac{\frac{\gamma_{W}(1\;+\;cos\;\theta_{W})}{2}\;-\;\sqrt{\gamma_{S}^{d}\gamma_{W}^{d}}}{\sqrt{\gamma_{W}^{p}}}\right]}^{2}$$

The total free energy of a surface is the sum of both components:

(5) $$\gamma_{S}\;=\;\gamma_{S}^{d}\;+\;\gamma_{S}^{p}$$

where

$\gamma_{S}^{d}$—dispersion component of the free energy of the surface of a solid.
$\gamma_{S}^{p}$—the polar component of the free energy of the surface of a solid.
$\gamma_{L}^{d}$, $\gamma_{L}^{p}$—dispersion and polar components of the surface tension of the liquid.
θ—measured wetting angle.

To evaluate the resistance of PHBV composites to a specific washing process, material samples were immersed in an aqueous detergent solution. The degradation medium was prepared using a commercial dishwasher detergent, Finish Classic Fresh/Lemon tablets (RB Hygiene Home Poland Sp. z o.o., Nowy Dwór Mazowiecki, Poland). According to the manufacturer’s safety data sheet, the formulation comprised sodium carbonate (50–75% by weight), disodium carbonate–hydrogen peroxide adduct (sodium percarbonate, ≤10% by weight), sodium silicate (≤1% by weight), and sodium salt of 1-hydroxyethylidene diphosphonic acid (≤1% by weight). Additionally, the detergent composition included 5–<15% by weight oxygen-based bleaching agents, <5% by weight phosphonates, <5% by weight non-ionic surfactants, <5% by weight polycarboxylates, as well as the enzymes subtilisin and amylase, and a fragrance composition. One tablet, with a measured mass of 16 g, was dissolved in 3 L of water, resulting in a concentration of 5.33 g/L. Each experimental vessel contained 10 specimens, and three independent vessels were utilized for each condition. The manufacturer states that the pH of a 10% product solution is 10.4; however, this value does not pertain to the exposure bath. This study investigates changes in mechanical properties during continuous exposure to the specified detergent solution at 60 °C. It does not specifically address hydrolytic, oxidative, enzymatic, or interfacial degradation mechanisms. Furthermore, continuous immersion does not replicate a cyclic household dishwashing protocol. The detergent solution was completely replaced every 7 days with a freshly prepared solution of the same concentration to limit changes in the chemical activity of the exposure medium during prolonged testing. Given that the detergent is a multicomponent commercial formulation containing alkaline, oxidizing, surfactant, chelating, and enzymatic constituents, the experiment should be considered as exposure to a complex detergent environment rather than a selective test of alkaline, oxidative, or enzymatic degradation. Following exposure, the samples underwent further characterization to identify changes induced by the detergent and elevated temperature. The study aimed to evaluate the chemical and thermal resistance of composites with a PHBV matrix under accelerated continuous exposure to a detergent solution used in automatic dishwashers. Special emphasis was placed on the potential degradation of the polyester matrix of PHBV due to increased temperature and the presence of alkaline, oxidising, and surfactant components in the dishwasher tablet. The objective was to ascertain whether such environmental exposure leads to alterations in the structure, morphology, and properties of the material.

Mechanical testing was conducted utilizing the Shimadzu AGS-X 10 kN testing machine (Kyoto, Japan). The Young’s modulus was determined using an Epsilon Model 3442 axial contact extensometer, which has a measurement range of 25 mm. The modulus was calculated over a strain range of 0.05% to 0.25% (Epsilon Technology, Jackson, USA). For the static tensile tests, standard multipurpose type A specimens were employed. These injection-moulded specimens, prepared in accordance with ISO 3167:2002 [24], featured a total length of 150 mm, a narrow parallel-section length of 80 mm, a narrow-section width of 10 mm, a grip-section width of 20 mm, a thickness of 4.0 mm, and a transition radius ranging from 20 to 25 mm. Tensile properties, such as tensile strength, Young’s modulus, and strain at break, were assessed following ISO 527 [25] at a crosshead speed of 10 mm/min. Tensile tests at elevated temperatures were conducted within an Instron Series 3119 (Instron, Norwood, MA, USA) environmental chamber. The samples were placed in a preheated chamber and conditioned for 15 min before testing to ensure uniform temperature distribution across each sample’s entire cross-section. Charpy impact strength was evaluated using a Zwick/Roell HIT5.5P pendulum impact tester (Ulm, Germany) equipped with a 5 J hammer, in accordance with ISO 179 [26]. Beam-shaped specimens, measuring 80 mm in length, 10 mm in width, and 4 mm in thickness, were utilized, resulting in a cross-sectional area of 40 mm^2^. The impact strength was calculated based on the absorbed impact energy relative to the specimen’s cross-sectional area. Formulas for calculating mechanical properties are presented in Equations (6)–(8).

Tensile strength (σt, MPa):

(6) $$\sigma_{t}=\frac{F_{max}}{A}\;$$

where

$F_{max}$—maximum force, N
A—initial cross-sectional area, ${mm}^{2}$

Strain at break (ε, %)

(7) $$\varepsilon =\frac{∆L}{L_{0}}\cdot 100\%$$

where

∆L—absolute elongation of the specimen, mm
$L_{0}$—initial gauge length, mm

Impact strength (I, kJ/m^2^)

(8) $$I=\frac{I_{u}}{A}\cdot 1000$$

where

$I_{u}$—impact energy required to fracture the specimen, J
A—initial cross-sectional area, ${mm}^{2}$

After excluding outliers, five measurements from the mechanical tests were used to calculate the arithmetic mean and standard deviation. For each formulation and set of test conditions, seven samples were tested, with the two most extreme results omitted from the statistical analysis.

Water absorption was assessed through an immersion method in accordance with ISO 62:2008 [27]. Before testing, the specimens were dried at a temperature of 50 ± 2 °C until a constant mass was achieved, then cooled to room temperature in a desiccator and weighed to ascertain the initial dry mass (W_0_). Subsequently, the specimens were fully immersed in distilled water maintained at 23 ± 1 °C. Water uptake measurements were conducted following immersion periods of 1, 10, 21, and 35 days. Mass measurements were carried out on five samples for each composition. At each specified interval, specimens were individually removed from the water, and excess surface water was carefully eliminated using dry filter paper. The specimens were weighed within one minute of removal from the immersion bath to determine the wet mass (Wn) and were then returned to the water. Periodic weighing of the specimens was performed using an electronic weighing balance (RADWAG WAS 22W,RADWAG Wagi Elektroniczne Witold Lewandowski, Radom, Poland). Water absorption was calculated using the following equation:

(9) $$\%W=\frac{W_{n}-W_{0}}{W_{0}}\cdot 100$$

where $W_{0}$ is the initial weight of the sample, $W_{n}\;$is the weight of the specimen after the specified immersion time, and %W is the percentage increase in weight.

Thermogravimetric analysis was performed using a TG 209 F3 Tarsus instrument (NETZSCH, Selb, Germany). Samples, each weighing approximately 10 mg, were placed in alumina crucibles and heated from 40 to 900 °C at a rate of 10 °C/min under a nitrogen atmosphere. The flow rates for the protective and sample purge gases were maintained at 10 and 20 mL/min, respectively. The term T5 refers to a 5% mass-loss threshold and should not be interpreted as the initial occurrence of chemical degradation. Mass normalization occurred at 20 °C. The TG/DTG plots presented in Section 3.4 show the recorded temperature program, while T_p_ indicates the temperature corresponding to the maximum mass-loss rate on the DTG curve. The residual mass fraction at 600 °C is denoted as R_600_.

### 2.3. Data Analysis

Results are presented as mean ± standard deviation, along with the actual number of independent specimens for each outcome and condition. Inferential comparisons are limited to outcomes where sample sizes, independence, and exclusions are verifiable from the experimental records. For these outcomes, Welch’s one-way ANOVA and Games–Howell pairwise comparisons are employed, with the tested comparison families and multiplicity correction detailed in the Supplementary Material. Outcomes lacking verified independent replication are interpreted descriptively, without assertions of statistical significance. Statistical significance was assessed at α = 0.05. Detailed ANOVA results and comparisons with neat PHBV are available in the Supplementary Material (Tables S1–S3).

## 3. Results and Discussion

### 3.1. Morphology of Fillers and Microstructure of Composites

**Figure 2:**
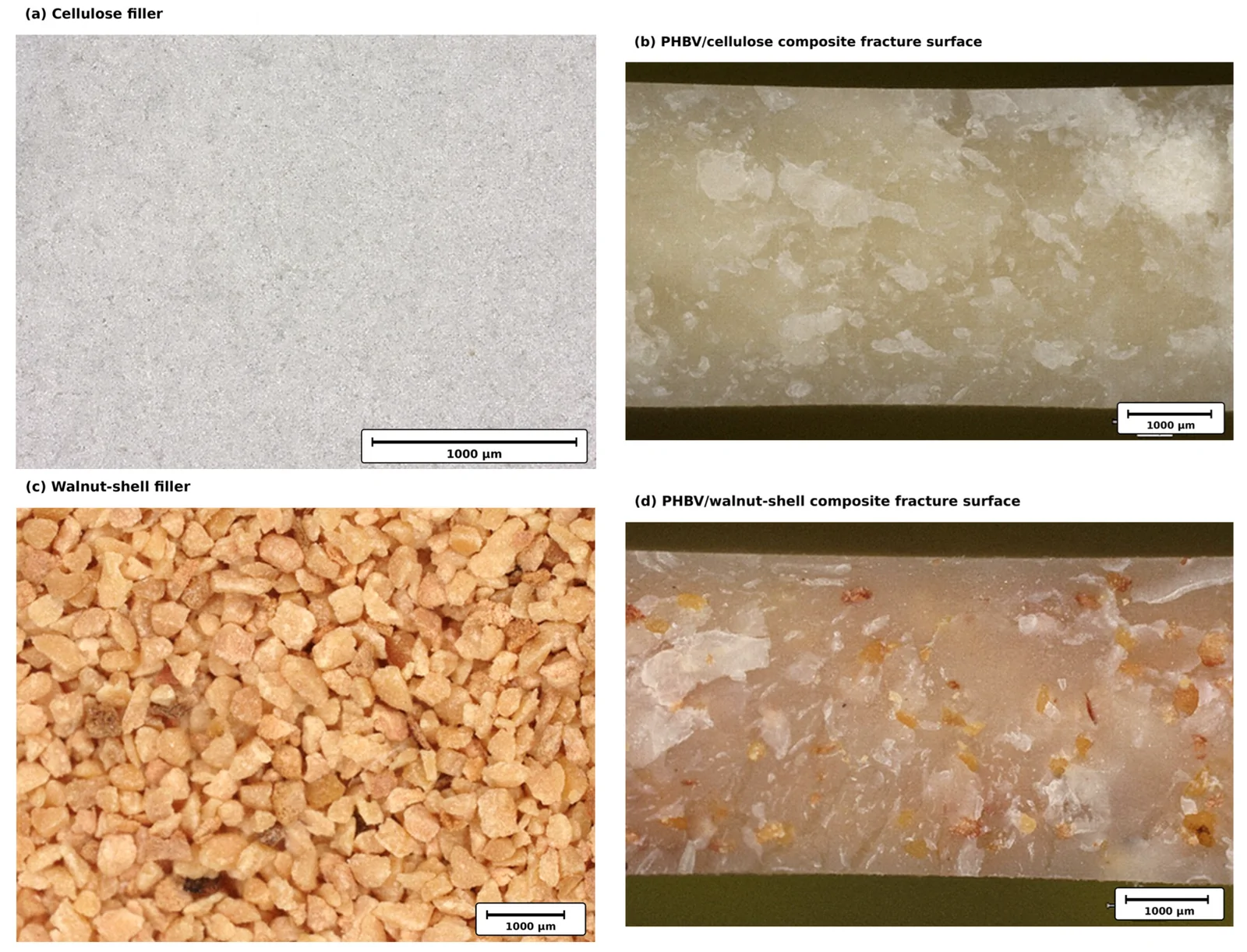

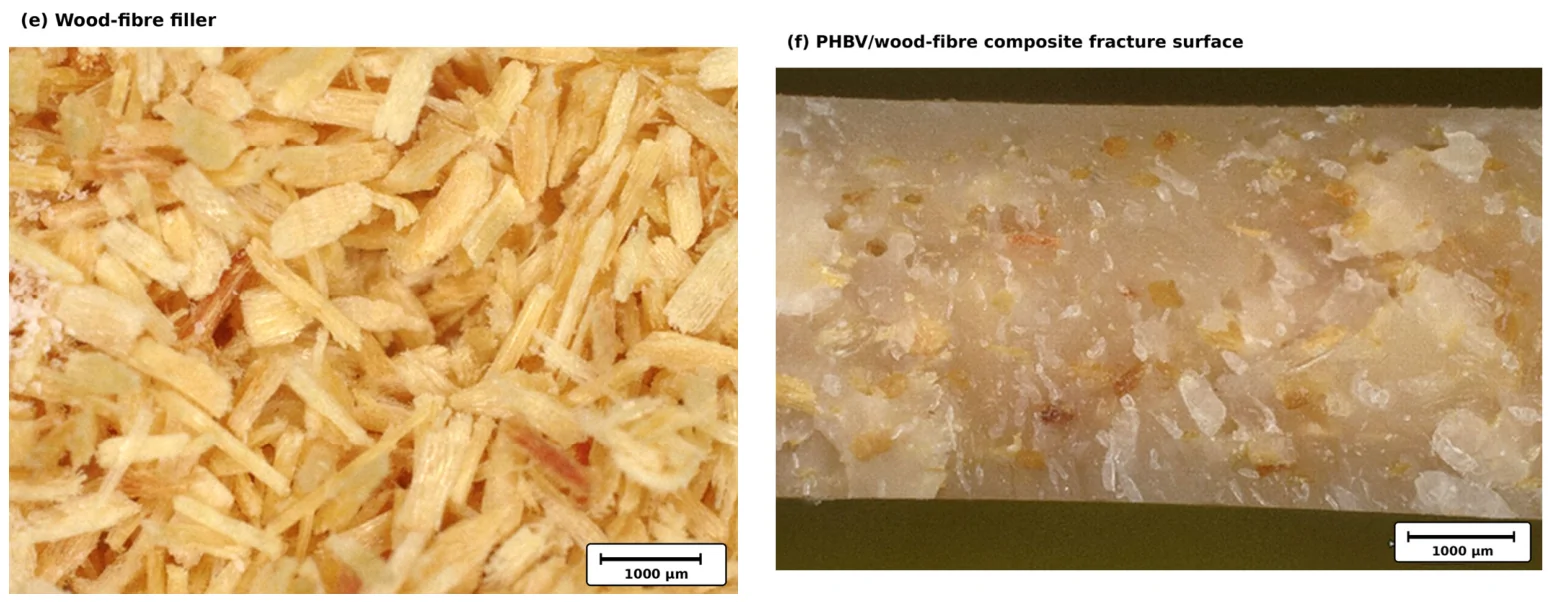
Optical micrographs of the natural fillers under investigation are presented in the left column: (**a**) cellulose; (**c**) walnut shell; (**e**) wood fibre, while the right column displays representative fracture surfaces of the corresponding PHBV composites with 20% by weight of fillers following tensile testing: (**b**) 20C; (**d**) 20R; (**f**) 20W.

**Figure 3:**
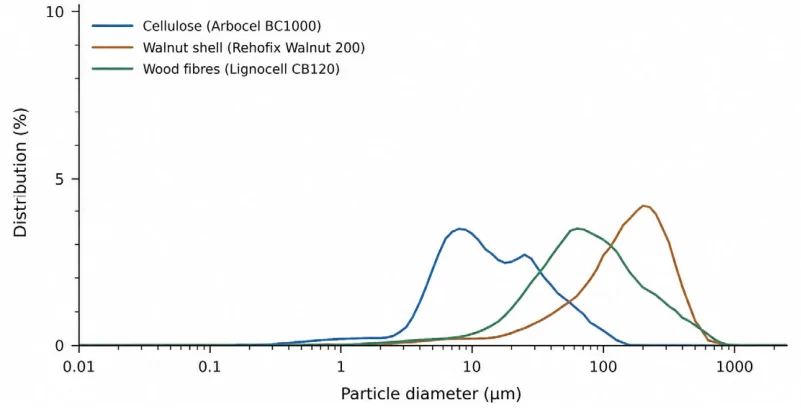
Particle size distributions of cellulose, walnut shells, and wood filler obtained by laser diffraction.

**Figure 4:**
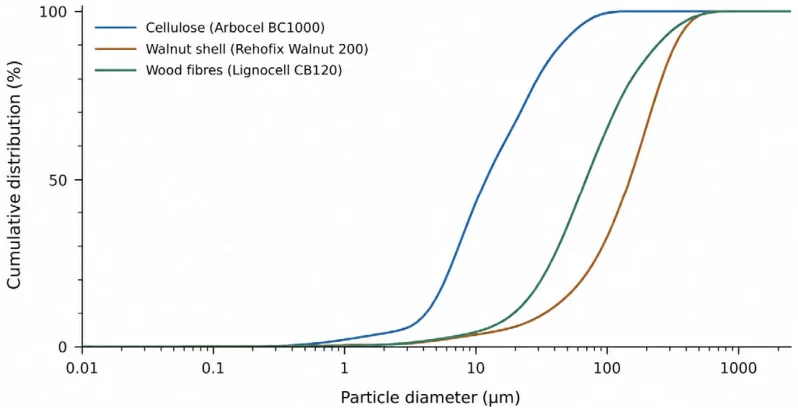
Cumulative particle size distributions of the investigated fillers.

Optical images of the fillers and composites (Figure 2) demonstrate morphological variations, including fine cellulose domains, irregular walnut shell particles, and elongated wood filler elements. Figure 3 and Figure 4 present the particle size distributions, with their parameters detailed in Table 3.

The median diameters (D50) are 11.9 µm for cellulose, 148.4 µm for walnut shells, and 69.1 µm for wood filler. The span values are 2.0 for walnut shells and 3.2 for wood, with the larger span for wood indicating a broader distribution of equivalent diameters. While these parameters characterize the variation in particle size, they do not quantify the fibre aspect ratio or the dispersion of filler in the final composite.

The image analysis results are summarised in Table 4. Cellulose showed the smallest resolved contrast domains. The majority of optically resolved contrast domains measured approximately 14–23 µm in diameter, with occasional larger clusters reaching approximately 45–60 µm. These measurements closely align with the nominal fibre diameter of approximately 20 µm as specified by the supplier. However, the nominal fibre length of 700 µm could not be reliably ascertained from the current micrograph due to fibre overlap and orientation. Walnut shell particles exhibited significantly larger and highly irregular projected dimensions. Image segmentation revealed a median apparent major-axis length of approximately 480 µm, with an interquartile range of approximately 345–676 µm and an approximate 5–95% range of 227–851 µm. These image-derived values surpass the supplier-reported particle-size range of 100–200 µm. Nonetheless, this discrepancy should not be construed as a contradiction of the manufacturer’s specification, as touching particles and local agglomerates are often segmented as single objects in two-dimensional optical images, thereby increasing the apparent projected size. Wood filler showed a clearly elongated morphology in the optical micrographs. Representative visible fibres had projected lengths of approximately 0.82–1.40 mm, with a median of about 0.99 mm, while their apparent widths were approximately 98–148 µm, with a median of about 115 µm. The measured apparent width falls numerically within the supplier-reported average particle-size range of 70–150 µm. However, it is important to note that these descriptors are not directly equivalent. Additionally, the significantly larger fibre lengths corroborate the elongated morphology of the wood filler. Overall, the optical observations confirm distinct morphological differences among the fillers: cellulose forms the finest dispersed phase, walnut shell consists of irregular particulate structures, and wood filler retains a distinctly elongated fibrous geometry.

**Figure 5:**
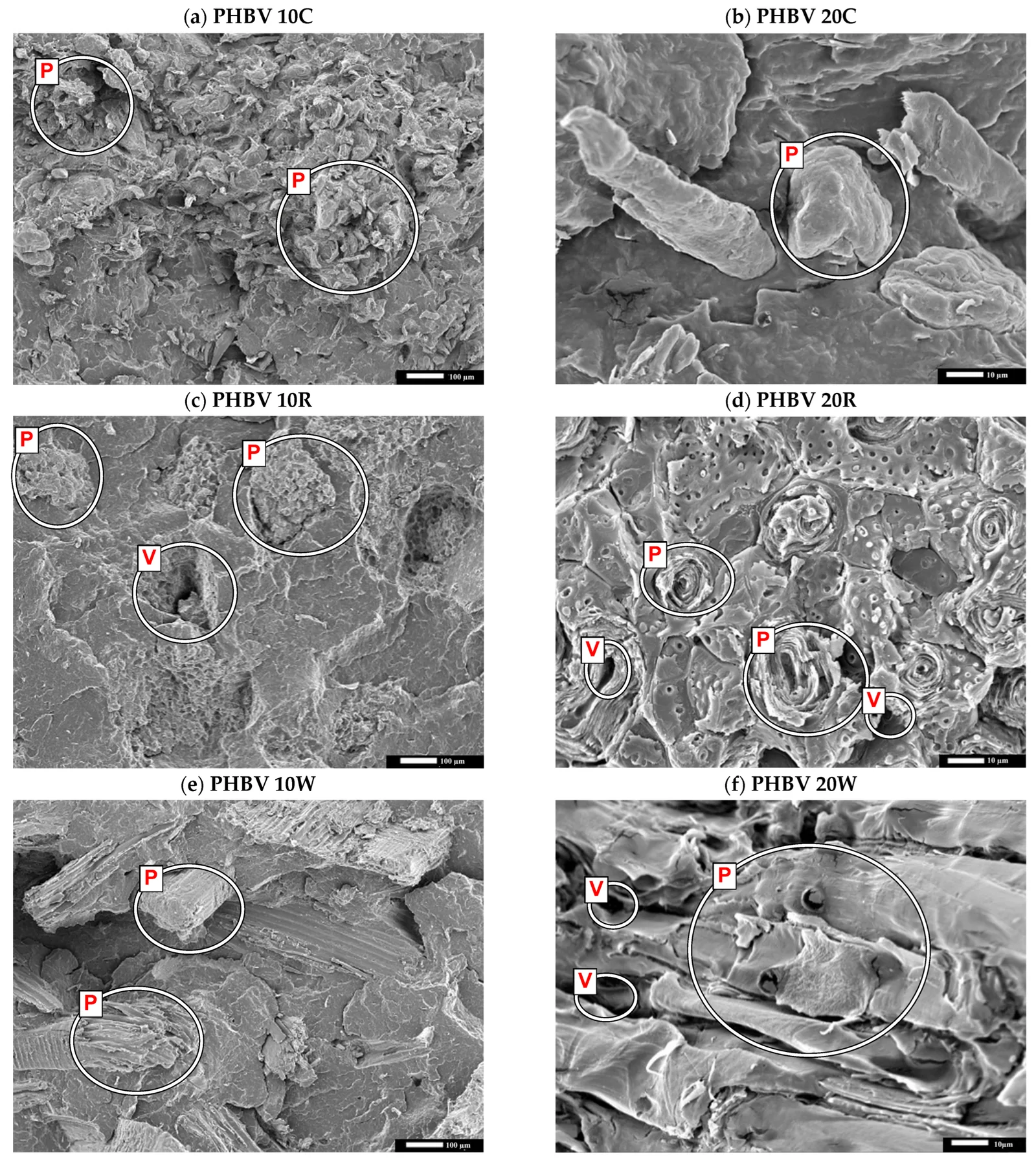
SEM images of tensile-fracture surfaces: (**a**) PHBV 10C, (**b**) PHBV 20C, (**c**) PHBV 10R, (**d**) PHBV 20R, (**e**) PHBV 10W, and (**f**) PHBV 20W. Markers P and V identify features interpreted as exposed filler and local voids, respectively. Scale bars are 100 µm in (**a**,**c**,**e**) and 10 µm in (**b**,**d**,**f**). The images support qualitative interpretation; defect densities are not compared quantitatively.

Figure 5 illustrates the morphology of the tensile fracture surface. The presence of local gaps, exposed particles, and indications of filler pull-out suggest that the interface contributes to the fracture process.

The local gaps and exposed filler features identified in the fracture micrographs align with interfacial debonding and particle pull-out during fracture. In nutshell-filled composites, the cavities observed are similarly indicative of these mechanisms. These morphological characteristics imply that interfacial failure may play a role in the fracture process. Nevertheless, the images offer only qualitative evidence and do not quantify filler–matrix adhesion, filler dispersion, specific interfacial area, or the relative contribution of each fracture mechanism [3,28,29].

In wood composites, the elongated fibre geometry facilitates efficient load transfer, while simultaneously emphasizing the significance of the fibre–PHBV interface quality. The presence of visible gaps and pull-out marks suggests that some fibres were extracted from the matrix rather than being torn. Research on PHBV reinforced with natural fibres indicates that enhancing fibre compatibility and wetting reduces pull-out and improves stress transfer efficiency [28,30]. At a concentration of 20% by weight, an increase in interfacial boundaries can concurrently elevate the proportion of the rigid phase and the number of potential damage initiation sites. This compromise between stiffening and structural defects has been similarly observed in PHBV composites with various plant fillers [3,31,32].

The composite densities presented in Table 2 range from 1.257 to 1.298 g/cm^3^, indicating an increase of approximately 0.6–3.8% compared to the density of PHBV, which is 1.250 g/cm^3^. An increase in filler content from 10 to 20% by weight results in a density increase of 0.002 g/cm^3^ for C, 0.023 g/cm^3^ for R, and 0.019 g/cm^3^ for W. The study by Mazur [19] provides a direct comparison of cellulose, walnut shells, and wood within a PHBV matrix, including a formulation with a filler content of 7.5% by weight.

### 3.2. Surface Properties and Wettability

The measurements of the wetting angle with water and diiodomethane, along with the calculated total free energy of the surface, are detailed in Table 5. For unfilled PHBV, the wetting angle with water was recorded at 76.65°. The addition of each filler resulted in a reduction in this angle: at 10% by weight, the angles decreased to 69.15° for PHBV 10C, 71.69° for PHBV 10R, and 64.14° for PHBV 10W. At 20% by weight, the angles further decreased to 63.18°, 63.42°, and 58.38°, respectively. Consequently, PHBV 20W exhibited the highest water wettability, with an angle approximately 24% lower than that of pure PHBV. The total free energy of the PHBV surface was initially 50.10, but for the 20% by weight variants, it increased to 55.20 (20C), 55.80 (20R), and 58.52 (20W). At 10% by weight, the changes were less pronounced and non-monotonic, with values of 49.54 for 10C, 50.72 for 10R, and 53.02 for 10W.

The reduction in the wetting angle following the introduction of the natural phase suggests an enhanced surface affinity of the composites for water. This observation aligns with the presence of hydroxyl groups in cellulose and hemicellulose, which augment the proportion of polar interactions on the composite surface [6,17,32,33,34]. The pronounced effect observed for wood filler may be attributed to the synergy between its surface chemistry and its fibrous, more developed morphology. Similar investigations of PHBV with natural fibres have demonstrated that chemical modification of the fibres concurrently alters the interface morphology, mechanical properties, and wetting angle, thereby confirming the strong interrelation between surface composition and topography [6].

The increase in total free surface energy at 20% by weight aligns with the changes observed in the wetting angle; however, the results at 10% by weight indicate that these two quantities do not necessarily change proportionally. The angle measurement for water is particularly sensitive to polar components, whereas the total surface energy, determined using multiple liquids, also depends on the dispersive component. Furthermore, the wetting angle is influenced by local roughness, heterogeneity, and the degree of exposure of filler particles. Nonetheless, the findings clearly demonstrate that increasing the lignocellulosic phase to 20% by weight enhances the surface hydrophilicity, most notably in the system containing wood.

### 3.3. Mechanical Properties at 23 and 80 °C

**Figure 6:**
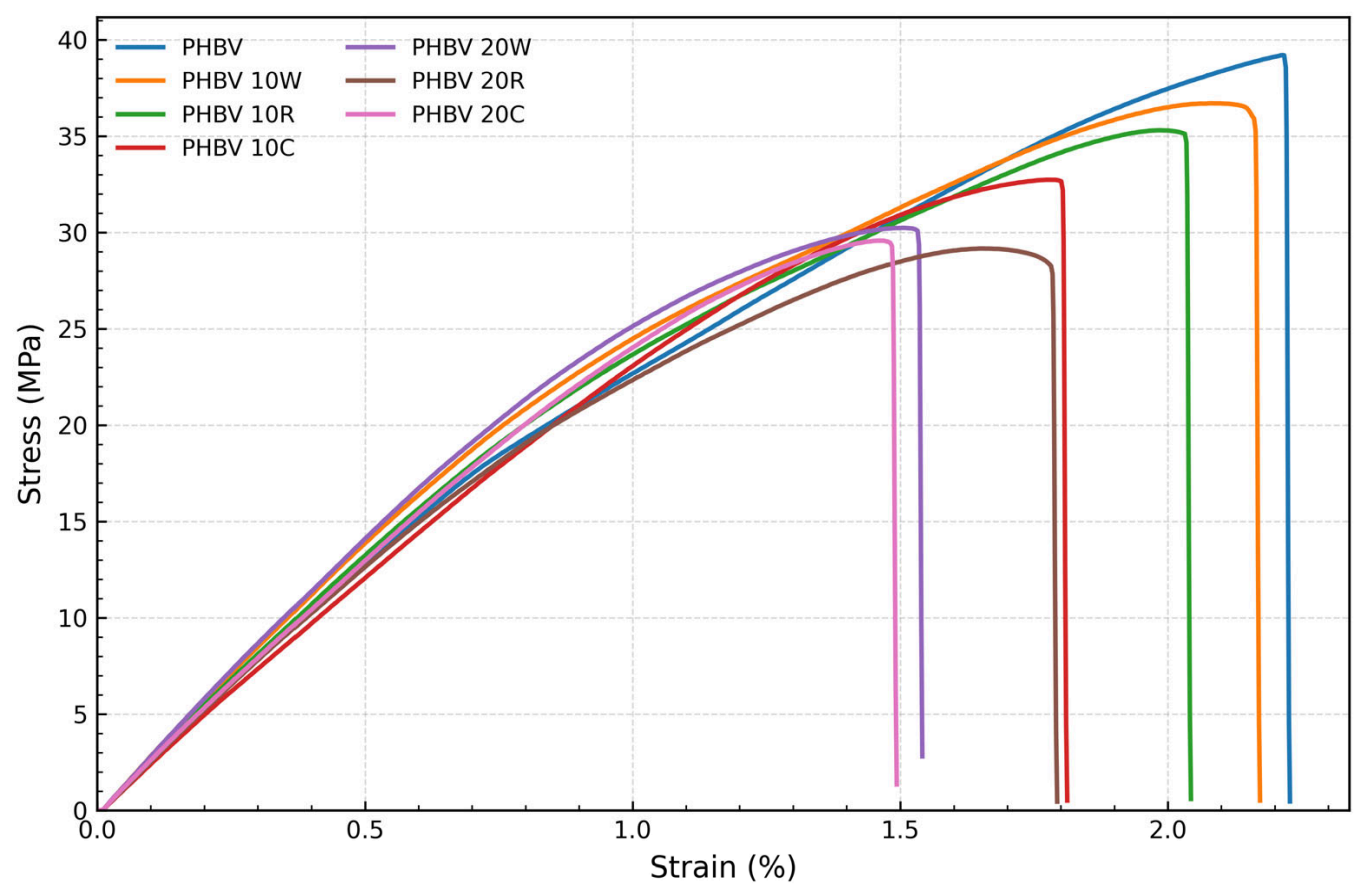
Representative tensile stress–strain curves.

**Figure 7:**
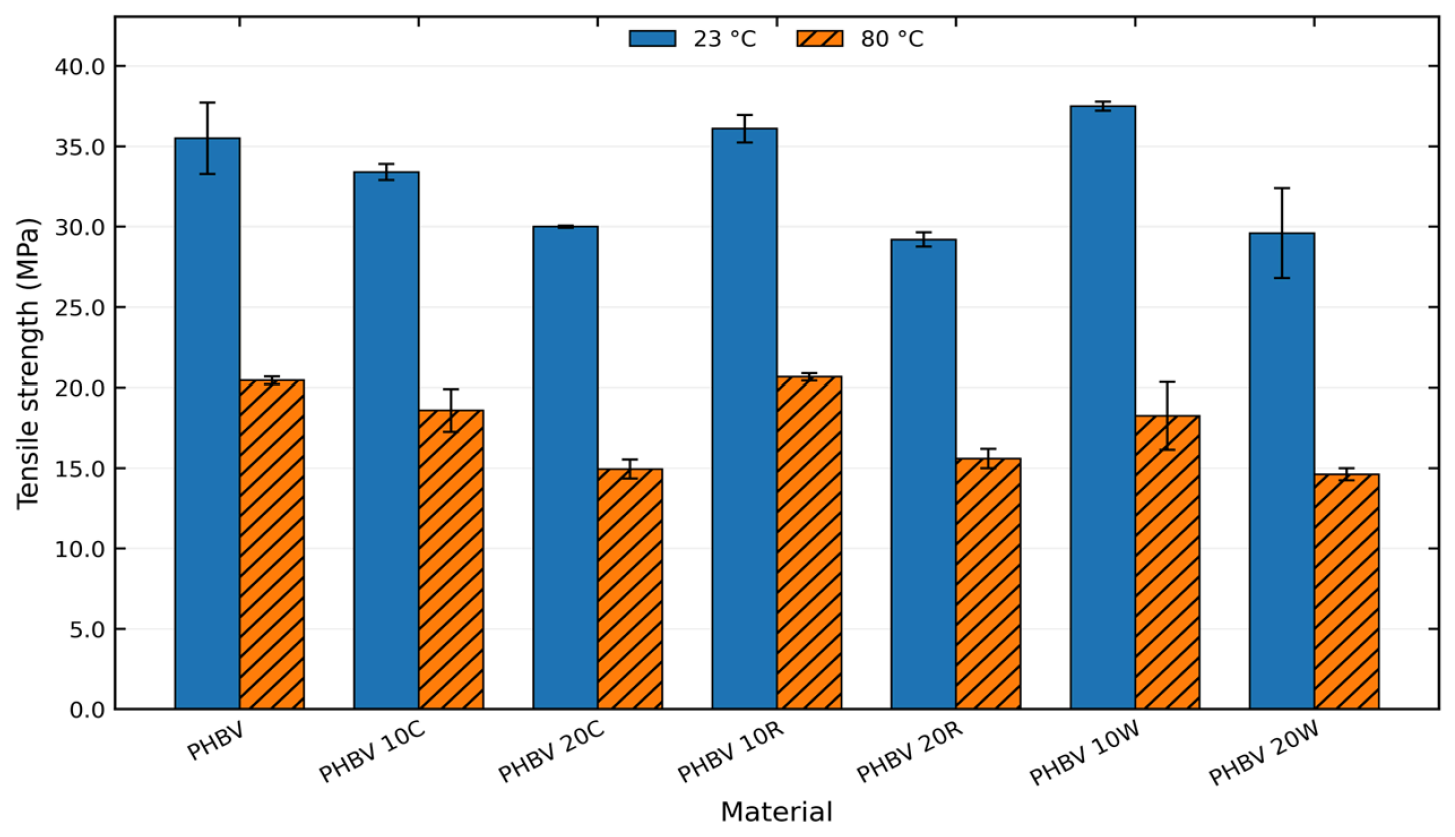
Tensile strength of PHBV and PHBV-based composites measured at 23 and 80 °C.

**Figure 8:**
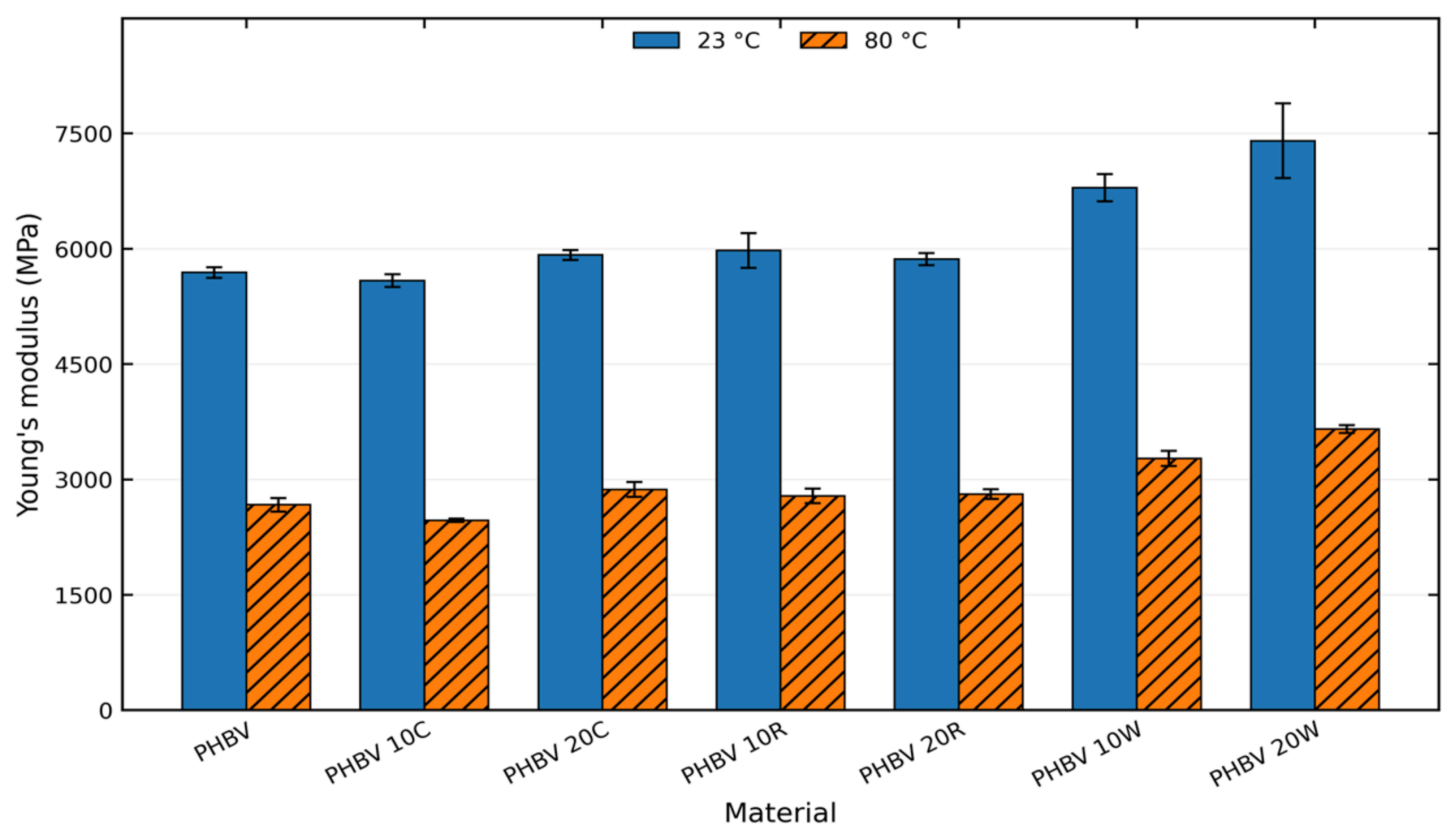
Young’s modulus of PHBV and PHBV-based composites measured at 23 and 80 °C.

**Figure 9:**
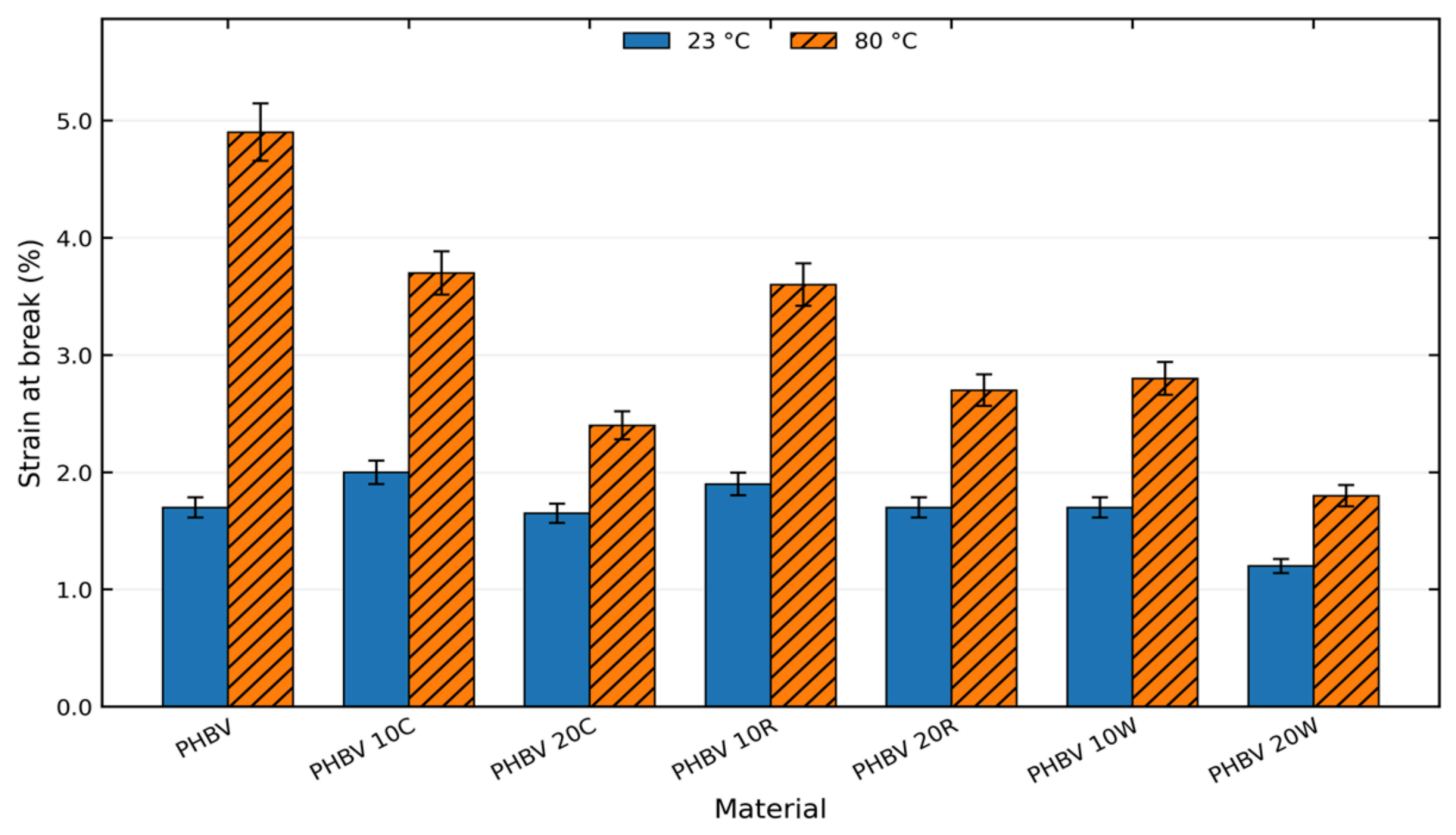
Strain at break of PHBV and PHBV-based composites measured at 23 and 80 °C.

The mechanical properties of the materials tested at room temperature are detailed in Table 6, while the representative tensile curves are illustrated in Figure 6. Comparative descriptive analyses of the values at 80 °C are provided in Figure 7, Figure 8 and Figure 9, which do not indicate statistically significant differences between the formulations.

At 23 °C, PHBV 10W exhibited the highest mean tensile strength at 37.5 MPa, representing only a 5.6% increase over PHBV. The modulus of the W series increased by 19.3% at 10% by weight and by 30.1% at 20% by weight compared to the matrix (Figure 7 and Figure 8). However, at 20% by weight, the tensile strength of all composites decreased by 15.5–17.7% relative to PHBV, and the elongation at break for 20W reduced to 1.2% (Figure 9). The differentiation between small-strain stiffness and strength at failure aligns with the effects of a rigid phase and local stress concentrators [4,12,35,36]. SEM images suggest that filler debonding and pull-out may contribute to the reduction in strength, though they are not the sole causes [3,4,35,36,37].

PHBV 10C demonstrated the highest impact strength at 8.9 kJ/m^2^, which is 89.4% greater than that of PHBV, despite a 1.8% lower modulus. At 20% by weight C, the impact strength decreased by 38.2% compared to 10C, suggesting that the increased energy absorption during dynamic impact is not solely due to increased stiffness. Potential energy dissipation mechanisms include crack path deflection and local particle debonding. The low elongation across all formulations indicates a limited capacity for deformation prior to fracture [12,35,36].

The significance of the cellulose preparation method was demonstrated by Mathel et al. [38]. For PHBV containing microfibrillated cellulose derived from sorghum, the MFC-PEB method enhanced strength and modulus by 20% and 22%, respectively, compared to composites with unprocessed filler. This underscores the importance of filler preparation and dispersion for reinforcement efficiency.

Elevating the test temperature to 80 °C resulted in a reduction in the tensile strength of all materials. The highest tensile strength values were recorded for PHBV 10R (20.67 MPa) and PHBV (20.45 MPa). In contrast, at a filler content of 20% by weight, the tensile strength was measured at 14.95 MPa for 20C, 15.60 MPa for 20R, and 14.62 MPa for 20W. Concurrently, wood composites maintained the highest modulus values: 3273 MPa for 10W and 3655 MPa for 20W, although these values were approximately half of those observed at 23 °C. Conversely, elongation at break increased to 4.9% for PHBV, 3.7% for 10C, 3.6% for 10R, and 2.8% for 10W, with 20% by weight variants achieving 1.8–2.7%. The response at 80 °C suggests that matrix temperature plays a more significant role than filler geometry in influencing strength. As temperature rises, PHBV becomes more prone to deformation, diminishing the stress transmitted by the matrix and limiting the efficiency of load transfer to the fibres. Although rigid wood fibres continue to enhance modulus, the weakened response of the matrix and interface prevents this effect from translating into high strength. This separation of modulus, strength, and deformation changes aligns with the thermomechanical behaviour of PHBV composites and observations of hydrothermal ageing of this polyester [12,35,36].

### 3.4. Thermal Decomposition

**Figure 10:**
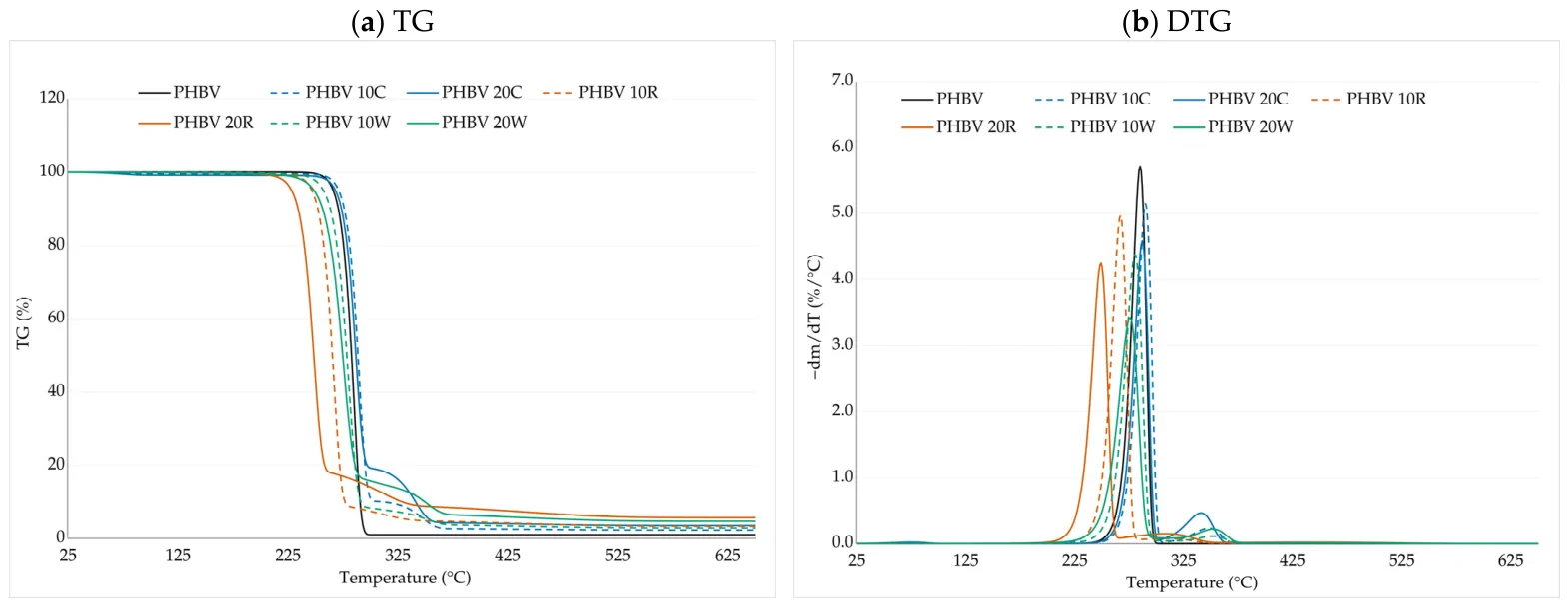
Thermogravimetric analysis of PHBV and the composites: (**a**) TG curves—relative mass; (**b**) DTG curves—mass-loss rate.

The TG and DTG curves are shown in Figure 10, and the characteristic temperatures and residue are listed in Table 7.

The T_5_ values demonstrate varying effects of the fillers. Specifically, for 10C and 20C, the T_5_ values are elevated by 5.0 and 1.9 °C, respectively, compared to PHBV. In contrast, the incorporation of walnut shells results in a T_5_ reduction of 20.0 °C at 10% by weight and 38.0 °C at 20% by weight, while the corresponding reductions for wood are 10.9 and 17.4 °C. Consequently, 20R exhibits the lowest T_5_ and T_p_ values. An increase in the contents of walnut shell and wood resulted in a reduction in the temperature at which the composite experienced a 5% mass loss. This threshold is separate from the initial point of detectable mass loss and does not indicate the absence of reactions at lower temperatures.

**Figure 11:**
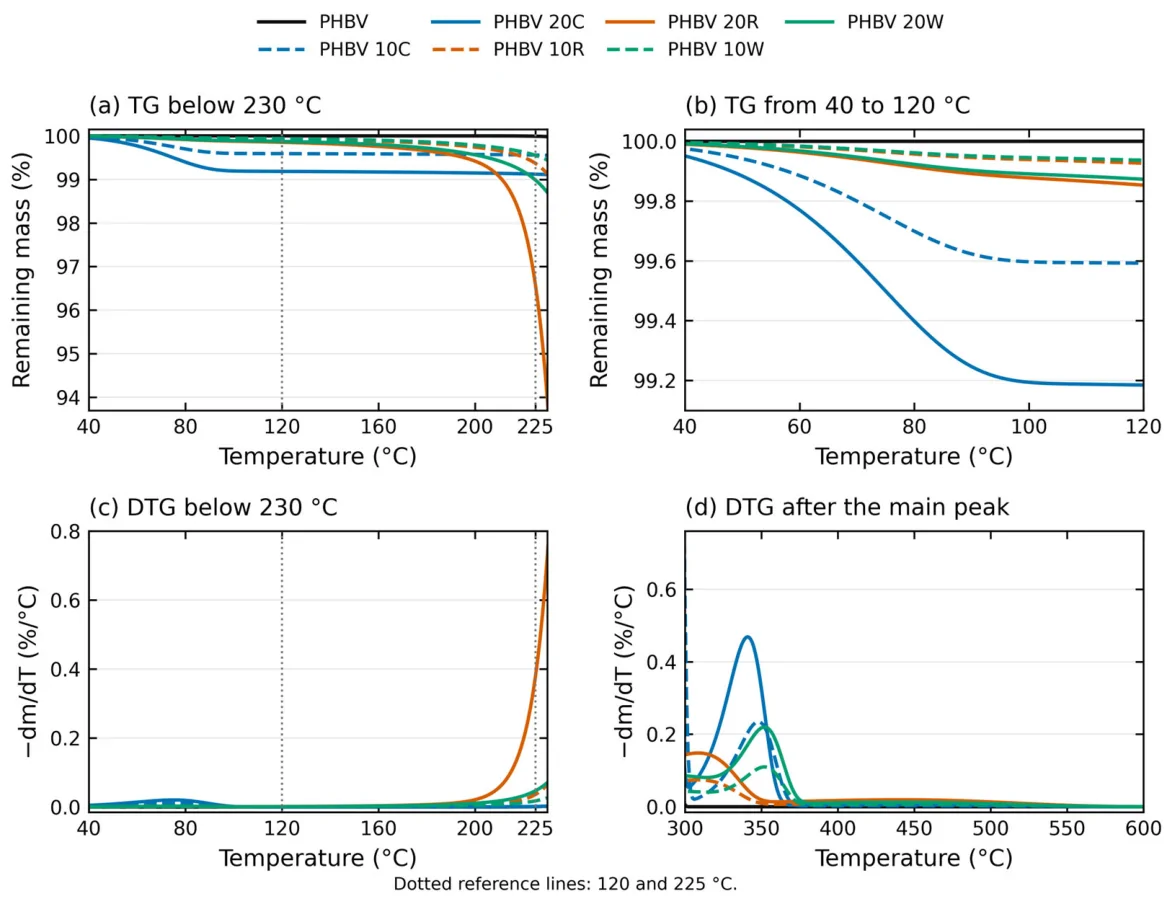
Magnified views of TG and DTG. (**a**) TG 40–230 °C; (**b**) TG 40–120 °C; (**c**) DTG 40–230 °C; (**d**) DTG 300–600 °C. Reference lines in panels (**a**,**c**): 120 and 225 °C.

The residual mass, R_600_, increases from 1.0% for PHBV to 3.6% for 20C, 5.8% for 20R, and 4.8% for 20W. Under nitrogen conditions, this residual mass represents the total residue from decomposition processes, potentially including charred organic matter and mineral constituents, and should not be solely equated with ash or residual PHBV. The higher residue and lower T_5_ for 20R are not contradictory, as the temperature at 5% mass loss and the amount of residue describe distinct aspects of decomposition. Santos et al. [39] similarly reported an earlier onset of mass loss in the PHBV/wood system, with the temperature at 2% mass loss decreasing from 261.6 to 232.6 °C. The detailed TG/DTG views are presented in Figure 11, with the mass losses over selected temperature intervals compiled in Table 8.

The magnified thermogravimetric (TG) curves indicate mass changes below 225 °C that are not easily discernible on the full-scale plot. The mass loss observed between 40 and 120 °C varied from less than 0.001% for neat PHBV to 0.766% for PHBV 20C. In the temperature range of 120 to 225 °C, the mass loss ranged from 0.008% for PHBV to 3.254% for PHBV 20R. For PHBV 20R, the cumulative mass loss at 225 °C was 3.391% relative to the initial mass at 25 °C. These findings suggest that the initial TG traces should not be characterized as constant. All interval losses were calculated as differences in normalized mass and expressed relative to the same initial mass. The attribution of these losses exclusively to water necessitates further supporting evidence.

The analysis of the low-temperature region must differentiate between moisture-related mass loss and the decomposition of organic components. According to Yang et al., the primary mass-loss intervals for isolated hemicellulose and cellulose occur at 220–315 °C and 315–400 °C, respectively, while lignin decomposes over a broader range of approximately 160–900 °C [40]. These intervals serve as a reference rather than definitive limits for PHBV composites. David et al. identified a principal degradation event for PHBV at 260–310 °C, with partially overlapping degradation of the matrix and filler in PHBV/cellulose composites [41]. Therefore, the presence of lignocellulosic filler alone does not necessarily indicate significant mass loss below 225 °C. The overlapping TG/DTG characteristics do not allow for the quantitative attribution of specific mass-loss fractions to individual components.

### 3.5. Water Absorption

**Figure 12:**
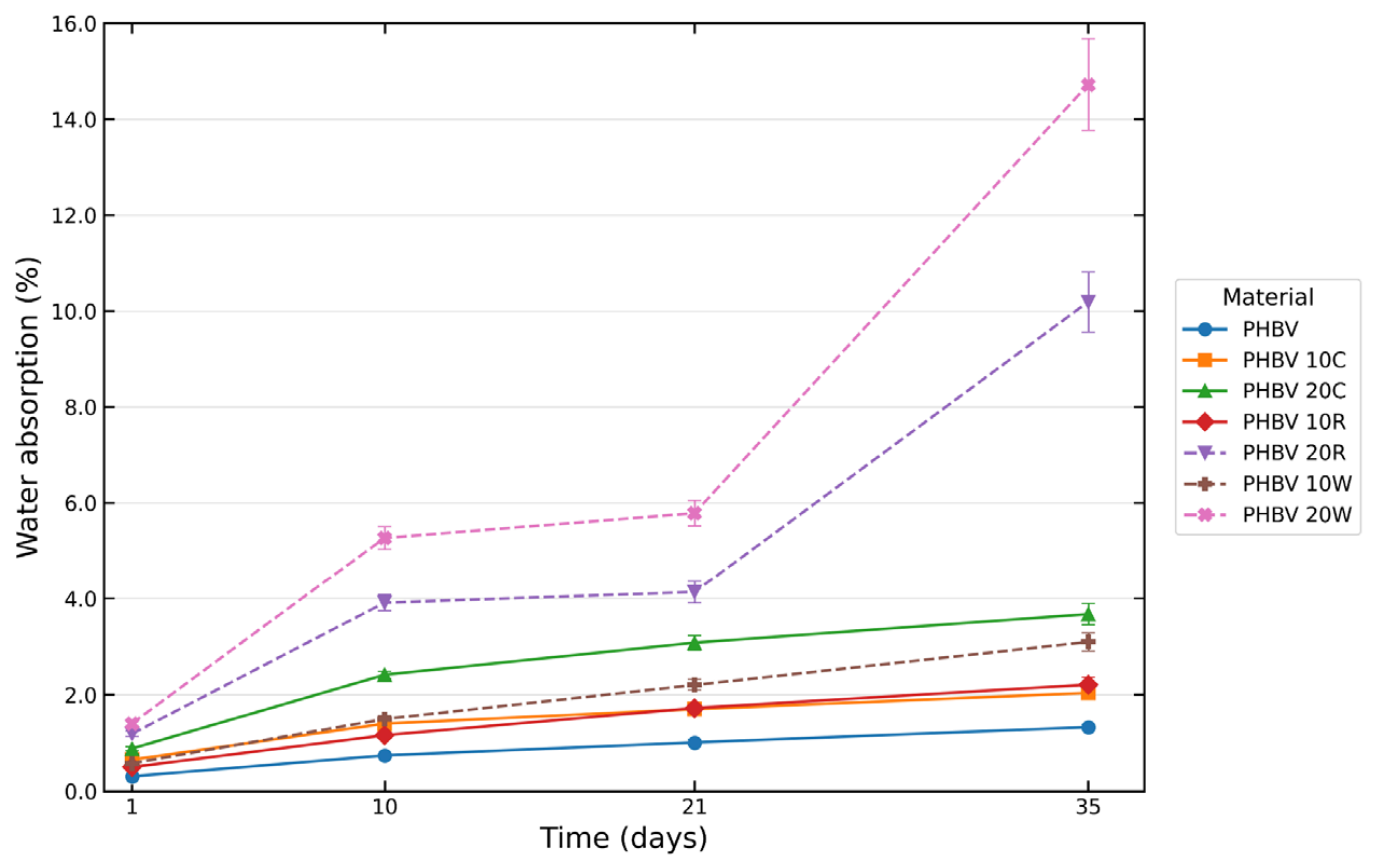
Water absorption of PHBV and PHBV-based composites as a function of immersion time up to 35 days.

Figure 12 illustrates the progression of water absorption. Unfilled PHBV exhibited the lowest weight gain at each analysed interval, with values ranging from 0.3% after 1 day to 1.3% after 35 days. The incorporation of fillers enhanced absorbency, with a pronounced effect at 20% by weight. After 35 days, water uptake values were 3.7% for PHBV 20C, 10.2% for PHBV 20R, and 14.7% for PHBV 20W. For the 10% by weight variants, the absorption rates were 2.0%, 2.2%, and 3.1%, respectively. Consequently, the 20W PHBV absorbed approximately 11 times more water than the unfilled PHBV, while the 20R absorbed about 7.8 times more. Notably, the increase in absorption between the 21st and 35th day was marked, rising from 5.8% to 14.7% for 20W and from 4.1% to 10.2% for 20R.

The observed modifications correspond to both surface characteristics and microstructure. Lignocellulose phases, which contain hydrophilic hydroxyl groups, demonstrate an increased number of water sorption sites as their proportion increases [31,42,43]. In wood composites, additional factors such as elongated and porous fibre morphology and an extended phase boundary may also play a role. PHBV 20W exhibits the lowest wetting angle, the highest surface energy, and the greatest gravimetric water uptake, indicating a material with a strong affinity for moisture. The pull-out traces and gaps observed in SEM suggest potential transport routes along the interface, and the swelling of the natural phase may exacerbate these discontinuities over time. The relationship between increased absorbency and filler content, as well as the enhancement of water resistance following improved phase compatibility, has been documented for both PHBV/cellulose and PHBV/fibres, and more broadly for lignocellulosic composites [12,27,41,42,43,44].

The reduced final absorption of cellulose composites does not necessarily indicate a decrease in the hydrophilicity of the cellulose itself. The finer phase, which is partially enveloped by the matrix, is more prone to forming less continuous transport pathways compared to a fibrous wood filler. Consequently, the differences observed among C, R, and W should be attributed to the interplay of factors such as chemistry, particle size and geometry, dispersion, and interface quality, rather than solely the presence of -OH groups. The rapid increase in sorption for 20R and 20W after 21 days implies a modification in transport conditions, potentially due to swelling and the formation of microcracks [41,42].

Janowski et al. [45] reported a comparable interdependence between increased stiffness and water absorption in PHBV containing sunflower seed hulls. At a filler content of 45% by weight, the modulus reached approximately 4.5 GPa, in contrast to 2.6 GPa for PHBV alone, while water absorption also rose. These consistent changes underscore the necessity of jointly assessing mechanical properties and sorption.

### 3.6. Changes in Mechanical Properties During Detergent Exposure

**Figure 13:**
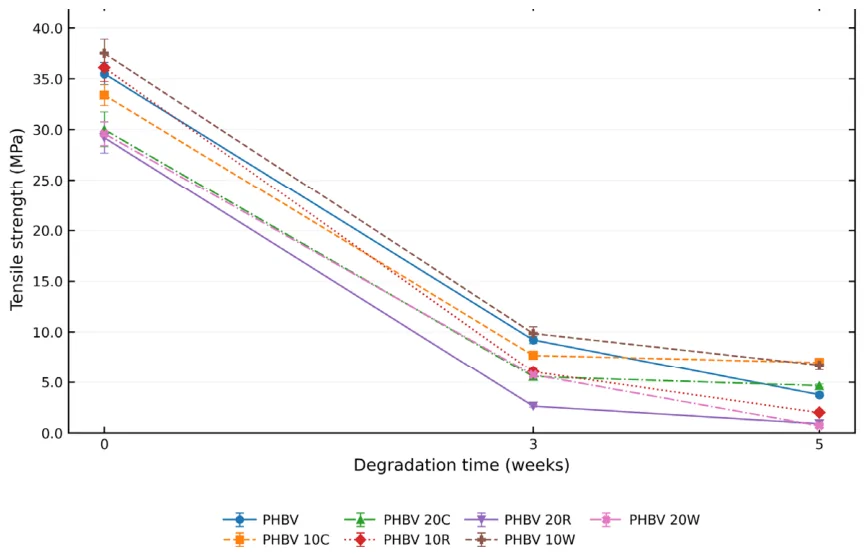
Tensile strength of PHBV and PHBV-based composites before exposure and after 3 and 5 weeks of detergent exposure.

**Figure 14:**
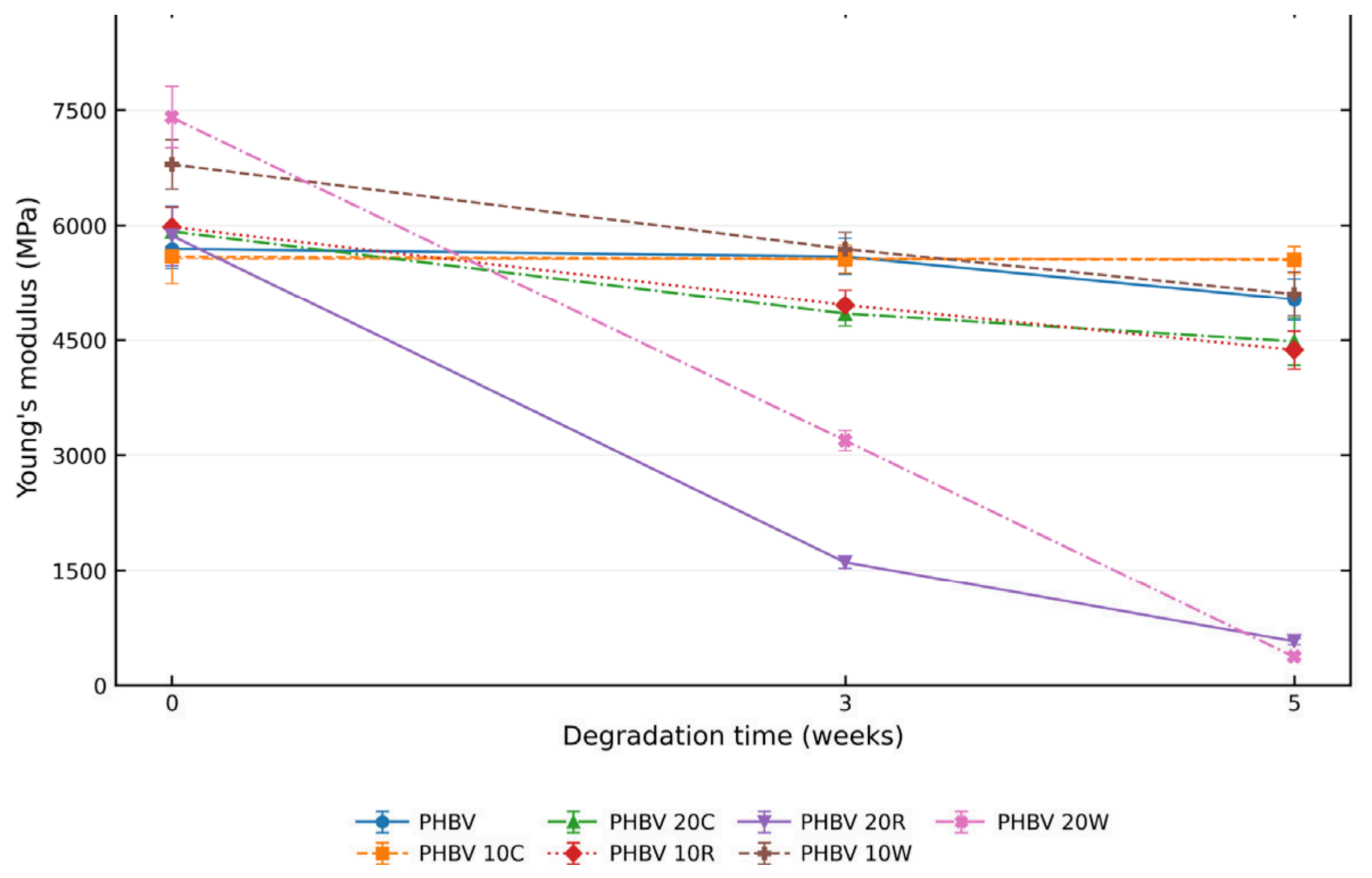
Young’s modulus of PHBV and PHBV-based composites before exposure and after 3 and 5 weeks of detergent exposure.

**Figure 15:**
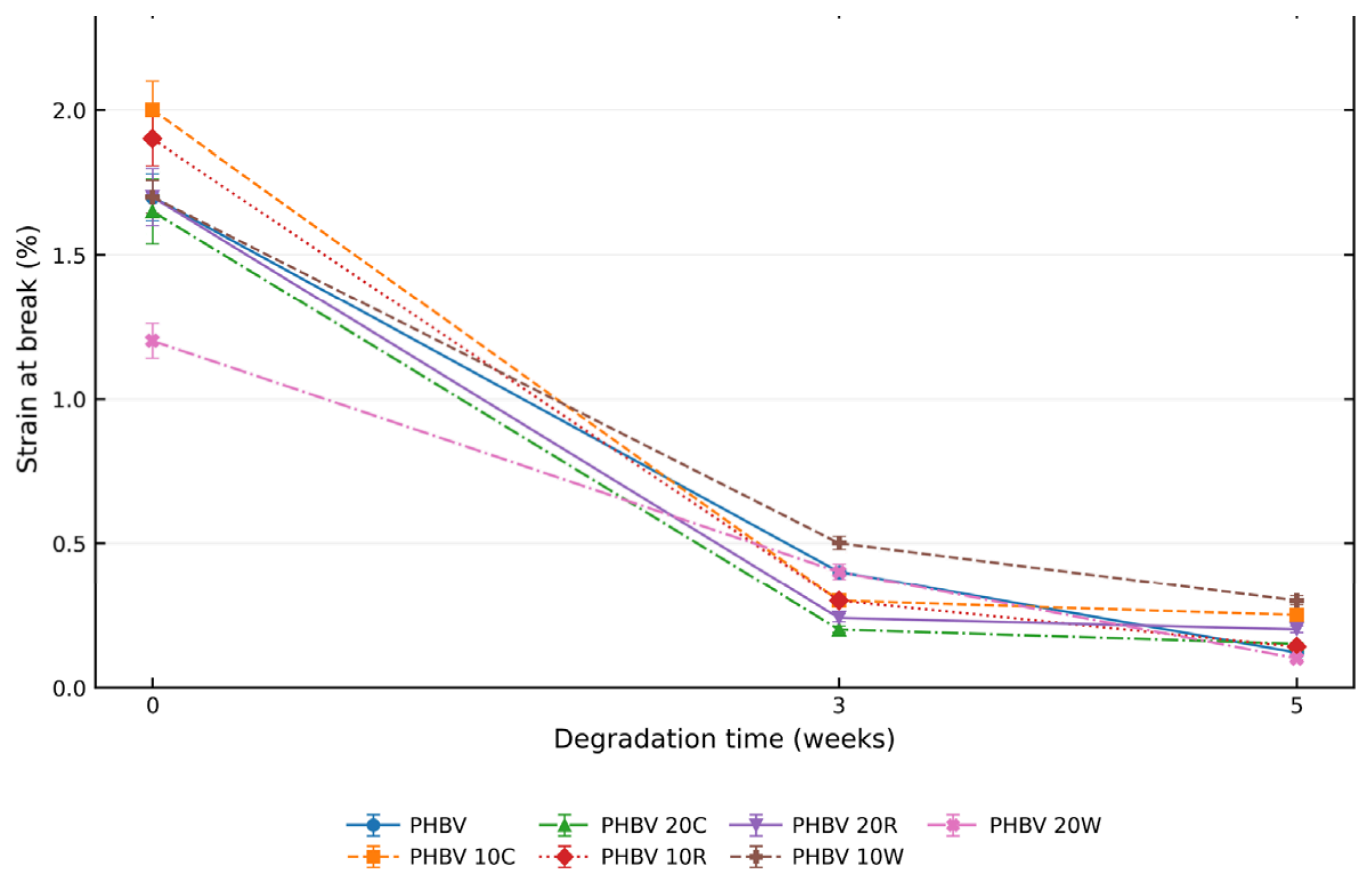
Strain at break of PHBV and PHBV-based composites before exposure and after 3 and 5 weeks of detergent exposure.

Figure 13, Figure 14 and Figure 15 illustrate the variations in strength, modulus, and elongation prior to exposure and following 3 and 5 weeks in the detergent solution, respectively.

Detergent exposure was accompanied by a marked reduction in mechanical properties, as illustrated in Figure 13, Figure 14 and Figure 15. After three weeks, a decline in tensile strength was observed across all materials. PHBV 10W and PHBV exhibited the highest tensile strengths, maintaining values of 9.8 MPa and 9.2 MPa, respectively, whereas PHBV 20R recorded a tensile strength of 2.6 MPa. By the fifth week, PHBV 10C and 10W retained tensile strengths of 6.9 MPa and 6.7 MPa, respectively, while 20R and 20W decreased to 0.9 MPa and 0.7 MPa, respectively. This indicates that PHBV 10W retained approximately 18% of its initial tensile strength, whereas 20R and 20W retained only about 3% and 2%, respectively. The changes in modulus were less uniform; after five weeks, PHBV maintained a modulus of 5030 MPa, 10C reached 5551 MPa, and 10W achieved 5100 MPa, while 20R and 20W decreased to 568 MPa and 368 MPa, respectively. For 20W, this represents a loss of approximately 95% of the initial modulus. The elongation at break diminished more rapidly and uniformly, reducing to approximately 0.2–0.5% after three weeks and 0.1–0.3% after five weeks. The comparisons of exposure times are descriptive in nature. They pertain to distinct tensile specimens that were tested prior to exposure and subsequently after 3 or 5 weeks. These comparisons alone do not establish statistical significance or identify a specific mechanism for the observed changes. Each formulation and time point involved testing 5 samples.

After five weeks, 10C retains approximately 99.4% of its initial modulus, yet only 20.7% of its initial strength. In comparison, the corresponding values for 10W are 75.1% and 17.9%, respectively, while the modulus of 20W decreases by approximately 95.0%. Thus, the retention of small-strain stiffness does not necessarily indicate the retention of load-bearing capacity up to failure. The comparability of the moduli after exposure is constrained by the low strain at break of certain specimens, which approaches the upper limit of the strain interval used to determine the modulus according to ISO 527. Potential contributions from filler swelling, interfacial changes, and PHBV hydrolysis align with the existing literature [12,46,47,48,49,50], although these factors were not experimentally isolated in this study.

The rate of change for individual parameters varies markedly. In the cases of PHBV, 10C, and 10W, both strength and elongation diminished prior to the modulus, indicating that the reduction in the capacity to withstand high stresses and deformations occurred before the complete loss of initial stiffness. In contrast, in 20R and 20W, there was significant water absorption and a marked decrease in rigidity when exposed to detergent. This observation corresponds with the progressive weakening of the extended filler–PHBV interface. Water has the potential to plasticize the available amorphous regions, while the swelling of the filler and the expansion of micro-gaps restrict load transfer. Additionally, prolonged exposure of PHBV to moisture at elevated temperatures may result in hydrolytic chain cleavage and a reduction in molar mass [12,46,47,48,49,50].

Abdallah and Ben Cheikh [51] also documented a discrepancy between the changes in modulus and the properties at break. In PHBV laminates incorporating nonwoven alfa fibres, both strength and elongation diminished by approximately 59% and 62%, respectively, following accelerated ageing. In contrast, the final modulus exhibited an increase of approximately 20% compared to the initial value. Existing literature indicates that PHBV is prone to hydrolysis in aqueous environments, with molecular changes being accelerated under hygrothermic conditions [46,47,48,49]. The current measurements demonstrate a reduction in mechanical properties when exposed to the specified multicomponent detergent solution. However, they do not determine whether hydrolysis, oxidation, crosslinking, or alterations in molar mass are responsible. Mechanisms suggested in the literature could potentially explain these observations, necessitating separate controls and chemical or molecular characterization for verification. Additionally, if the detergent used includes an oxidizer, radical reactions may further modify PHBV. Peroxide sources suggest that both coupling/crosslinking and chain-breaking reactions can occur, contingent upon the formulation [36,41,50]. Consequently, the present experiment should be regarded as an assessment of resistance to a complex aquachemical environment, rather than a definitive test of alkaline or oxidative degradation.

### 3.7. Synthesis of Observations and Limitations of Interpretation

The comprehensive analysis of the results reveals that the type of filler influences not merely a single parameter, but the entire sequence of structure-surface-mechanical-sorption relationships. Wood is the most effective stiffening agent; however, when incorporated at a concentration of 20% by weight, the composite exhibited the highest water absorption and significant reductions in mechanical properties upon exposure to detergent. Conversely, cellulose, at 10% by weight, offers reduced stiffness but improves impact strength and largely maintains its initial modulus, despite experiencing a considerable decrease in tensile strength during detergent exposure. The walnut shell filler occupies an intermediate position: at 10% by weight, it sustains high strength at 80 °C, whereas at 20% by weight, it demonstrates high absorption and significant deterioration in rigidity. Table 9 presents a summary of the key observations and the proposed mechanistic hypotheses.

## 4. Conclusions

The findings indicate that the properties of PHBV composites are influenced by the type and content of lignocellulosic filler under consistent processing conditions. At 23 °C, the average Young’s modulus increased from 5690 MPa for unfilled PHBV to 6791 MPa for PHBV 10W and 7403 MPa for PHBV 20W. PHBV 10W exhibited the highest average tensile strength (37.5 MPa); however, the Games–Howell comparison did not reveal a statistically significant increase compared to unfilled PHBV (35.5 MPa; p = 0.5107). All composites with 20% by weight filler demonstrated lower average tensile strength than PHBV. PHBV 10C showed the highest average impact strength (8.9 kJ/m^2^) compared to 4.7 kJ/m^2^ for PHBV.

All composite formulations displayed reduced water contact angles relative to PHBV, with PHBV 20W exhibiting the lowest angle (58.38°) and the highest total surface free energy (58.52 mJ/m^2^). After 35 days of immersion in distilled water at 23 °C, water absorption was measured at 1.3% for PHBV, 3.7% for PHBV 20C, 10.2% for PHBV 20R, and 14.7% for PHBV 20W. At 80 °C, both tensile strength and Young’s modulus decreased for all materials compared to their values at 23 °C. The temperature at 5% mass loss (T_5_) ranged from 229.2 °C for PHBV 20R to 272.2 °C for PHBV 10C, in contrast to 267.2 °C for PHBV; T_5_ should not be construed as the onset of decomposition.

Five weeks of continuous immersion in the specified detergent solution at 60 °C resulted in reductions in tensile strength and elongation at break across all formulations examined. PHBV 20R and PHBV 20W exhibited the lowest average tensile strengths post-exposure, approximately 0.9 and 0.7 MPa, respectively. These formulations also showed significant reductions in Young’s modulus. PHBV 10C retained approximately 99.4% of its initial average modulus but only 20.7% of its initial average tensile strength. Consequently, the retention of small-strain stiffness did not equate to the retention of tensile strength. The modulus values obtained for specimens with very low elongation at break necessitate caution due to the strain interval used for modulus determination.

Among the compositions examined, PHBV 10C exhibited the highest mean impact strength while demonstrating lower water absorption over 35 days compared to PHBV 20R and PHBV 20W. However, these findings do not determine an optimal filler content nor confirm suitability for repeated dishwashing cycles. The results align with the influence of filler morphology and interfacial characteristics, although these factors were not independently quantified. The continuous exposure to detergent did not allow for the isolation of the effects of water, temperature, and individual detergent components. Furthermore, the observed reductions in mechanical properties do not confirm biodegradation or a specific chemical degradation mechanism. To differentiate these effects, additional chemical and molecular analyses would be necessary.

## Figures and Tables

**Table 1:** Basic physical and mechanical properties of the PHBV-grade ENMAT Y1000P according to the manufacturer [20,21].

Density (g/cm^3^)	1.25
Melt flow rate (g/10 min)	5–15
Yield strength (MPa)	31–36
Tensile strength (MPa)	39
Elongation at break (%)	2
Elastic modulus (MPa)	2800–3500
Flexural modulus (MPa)	3520–4170
Vicat softening temperature (°C)	166
Melting point (DSC) (°C)	170–176

**Table 2:** Sample designations, filler characteristics, and measured densities of the investigated PHBV-based materials.

Index	Composite Composition	Density (g/cm^3^)	Moisture Content (% by Weight)
PHBV	Enmat Y1000P, PHBV Helian Polymers, China	1.250 ± 0.001	0.025%
Arbocel BC 1000 (C)	Arbocel BC 1000, Kremer Pigmente, Germany, length 700 µm, diameter 20 µm	1.5 (manufacturer’s data)	≤0.5
Rehofix, Walnut 200 (R)	Rehofix, Walnut 200, Rettenmaier, Germany, average particle size 100–200 µm	1.2–1.35 (manufacturer’s data)	≤0.5
Lignocell CB120 (W)	Lignocell CB120, Rettenmaier, Germany, average particle size 70–150 µm	1.5 (manufacturer’s data)	≤0.5
PHBV 10C	90% by weight PHBV + 10% by weight Arbocel BC 1000	1.296 ± 0.003	≤0.081
PHBV 20C	80% by weight PHBV + 20% by weight Arbocel BC 1000	1.298 ± 0.005	≤0.118
PHBV 10R	90% by weight PHBV + 10% by weight Rehofix, Walnut 200	1.259 ± 0.001	≤0.075
PHBV 20R	80% by weight PHBV + 20% by weight Rehofix, Walnut 200	1.282 ± 0.005	≤0.127
PHBV 10W	90% by weight PHBV + 10% by weight Lignocell CB120	1.257 ± 0.005	≤0.067
PHBV 20W	80% by weight PHBV + 20% by weight Lignocell CB120	1.276 ±0.002	≤0.135

**Table 3:** Volume-based equivalent-diameter distribution parameters of the fillers.

Material	D10 (µm)	D50 (µm)	D90 (µm)	Mean Size (µm)	Span (−)
Cellulose	4.2	11.9 ± 0.15	44.4 ± 1.94	22.0 ± 0.51	3.4 ± 0.12
Walnut shell	33.6	148.4 ± 1.20	328.6 ± 2.84	185.0 ± 1.22	2.0 ± 0.02
Wood fiber	19.5	69.1 ± 0.87	240.7 ± 6.69	110.0 ± 3.03	3.2 ± 0.07

**Table 4:** Semi-quantitative comparison of filler dimensions determined from optical micrographs with nominal dimensions reported by the suppliers.

Filler	Image Parameter	Image Analysis Result	Supplier Data	Interpretation
Cellulose (Arbocel BC1000)	Apparent resolved contrast-domain diameter	~14–23 µm; occasional larger clusters ~45–60 µm	Length ~700 µm; diameter ~20 µm	The resolved domains align with the nominal fibre diameter. However, the complete fibre length cannot be accurately determined from the available image.
Walnut shell (Rehofix Walnut 200)	Projected major-axis length of segmented objects	Median ~480 µm; IQR ~345–676 µm; approx. 5–95% range 227–851 µm	Average particle size 100–200 µm	The observed size exceeds the supplier’s specified value may partly reflect the segmentation of touching particles and agglomerates as single objects
Wood fibres (Lignocell CB120)	Apparent fibre width	Median ~115 µm; representative range ~98–148 µm	Average particle size 70–150 µm	The measured widths fall numerically within the size range reported by the supplier; however, the descriptors do not correspond directly.

**Table 5:** Contact angles with water and diiodomethane, along with the components and total surface energy of PHBV and its composites. Here, γd represents the dispersive component, γp denotes the polar component, and γ is the sum of these components.

Material	θ Water (°)	θ Diiodomethane (°)	γd (mJ/m^2^)	γp (mJ/m^2^)	γ (mJ/m^2^)
PHBV	76.7 ± 0.2	22.9 ± 0.1	46.9 ± 0.2	3.23 ± 0.2	50.1 ± 0.3
PHBV 10C	69.2 ± 0.1	33.9 ± 0.1	42.5 ± 0.6	7.01 ± 0.6	49.5 ± 0.7
PHBV 20C	63.2 ± 0.3	23.8 ± 0.2	46.6 ± 0.2	8.61 ± 0.3	55.2 ± 0.3
PHBV 10R	71.7 ± 0.2	26.8 ± 0.2	45.5 ± 0.4	5.24 ± 0.4	50.7 ± 0.5
PHBV 20R	63.4 ± 0.2	20.7 ± 0.2	47.6 ± 0.2	8.23 ± 0.3	55.8 ± 0.3
PHBV 10W	64.1 ± 0.2	30.1 ± 0.1	44.2 ± 0.2	8.85 ± 0.3	53.0 ± 0.3
PHBV 20W	58.4 ± 0.2	19.1 ± 0.3	48.0 ± 0.1	10.49 ± 0.2	58.5 ± 0.2

**Table 6:** Mechanical properties of PHBV and the composites at 23 °C, expressed as mean ± standard deviation. σt—tensile strength, Et—elastic modulus, ε—elongation at break, and I—Charpy impact strength.

Material	σt (MPa)	Et (MPa)	ε (%)	I (kJ/m^2^)
PHBV	35.5 ± 2.2	5690 ± 70	1.7 ± 0.2	4.7 ± 0.1
PHBV 10C	33.4 ± 0.5	5586 ± 83	2.0 ± 0.2	8.9 ± 0.2
PHBV 20C	30.0 ± 0.1	5918 ± 64	1.7 ± 0.1	5.5 ± 0.1
PHBV 10R	36.1 ± 0.9	5977 ± 226	1.9 ± 0.2	6.4 ± 0.4
PHBV 20R	29.2 ± 0.4	5865 ± 78	1.7 ± 0.2	5.1 ± 0.2
PHBV 10W	37.5 ± 0.3	6791 ± 177	1.7 ± 0.2	5.3 ± 0.2
PHBV 20W	29.6 ± 1.1	7403 ± 486	1.2 ± 0.2	4.1 ± 0.5

**Table 7:** TGA parameters: T_5_—temperature at 5% loss of the initial mass, T_p_—temperature at the maximum mass-loss rate, and R_600_—mass remaining at 600 °C.

Material	T_5_ (°C)	T_p_ (°C)	R_600_ (%)
PHBV	267.2	284.0	1.0
PHBV 10C	272.2	291.0	2.3
PHBV 20C	269.1	286.0	3.6
PHBV 10R	247.2	268.0	3.4
PHBV 20R	229.2	248.0	5.8
PHBV 10W	256.3	281.0	2.9
PHBV 20W	249.8	277.0	4.8

**Table 8:** Mass loss (Δm) of PHBV and PHBV-based composites over selected temperature intervals, expressed as a percentage of the initial mass at 25 °C.

Material	Δm 40–120 °C	Δm 120–225 °C	Δm 225–400 °C	Δm 400–600 °C	Δm 40–225 °C
PHBV	0.000	0.008	98.992	0.000	0.008
PHBV 10C	0.383	0.032	96.931	0.329	0.415
PHBV 20C	0.766	0.063	94.864	0.657	0.829
PHBV 10R	0.068	0.556	94.825	1.144	0.624
PHBV 20R	0.137	3.254	88.508	2.288	3.391
PHBV 10W	0.059	0.383	95.912	0.741	0.442
PHBV 20W	0.119	0.884	92.705	1.482	1.003

The Δm values are given as a percentage of the initial mass at 25 °C: Δmₐ–ᵦ = m(a) − m(b). They do not represent a percentage of the mass at the beginning of a given interval. A displayed value of 0.000 indicates rounding, not the detection limit.

**Table 9:** Synthesis of the main experimental observations and working hypotheses concerning mechanisms responsible for the observed trends.

Material/Additive	The Most Important Experimental Observation	Hypothetical Mechanism
PHBV	Lowest absorbency; decrease in strength and elongation during degradation with a relatively longer preserved modulus.	Degradation of the PHBV matrix itself; chain breakage and changes in the structure of the amorphous/crystalline phase.
C—cellulose	The absorbency exhibited a moderate increase. The highest mean impact strength was observed at 10% by weight, accompanied by a slightly lower initial modulus compared to PHBV.	High polarity but fine dispersion and low form factor; possible agglomeration. Fine particles can limit the size of macro-gaps despite water sorption.
R—walnut shell	10R retains good strength at 80 °C; 20R strongly absorbs water in the final phase and loses modulus after detergent exposure.	Thicker particles, stress concentration and local pull-out; progressive decoupling of the interface creating water penetration pathways.
W—wood	The largest initial modulus; 20W highest absorbency and very strong modulus loss after detergent exposure.	Fibrillar geometry provides stiffness, but hydrophilic and porous fibres and an extensive phase boundary increase sorption, swelling, and interface degradation.

## Data Availability

The original contributions presented in this study are included in the article/Supplementary Materials. Further inquiries can be directed to the corresponding author.

## Supplementary Materials

The following supporting information can be downloaded at: https://www.mdpi.com/article/10.3390/ma19214638/s1, Table S1. Welch’s ANOVA; df1 = 6. Significance is based on Holm-adjusted p values; Table S2. Mechanical properties at 23 °C and density; Table S3. Contact angles and surface energy.

## References

[1] Meereboer K.W., Misra M., Mohanty A.K. (2020). Review of recent advances in the biodegradability of polyhydroxyalkanoate (PHA) bioplastics and their composites. Green Chem..

[2] Zytner P., Pal A.K., Wu F., Rodriguez-Uribe A., Mohanty A.K., Misra M. (2023). Morphology and performance relationship studies on poly(3-hydroxybutyrate-co-3-hydroxyvalerate)/poly(butylene adipate-co-terephthalate)-based biodegradable blends. ACS Omega.

[3] Kuciel S., Mazur K., Jakubowska P. (2019). Novel biorenewable composites based on poly(3-hydroxybutyrate-co-3-hydroxyvalerate) with natural fillers. J. Polym. Environ..

[4] Frącz W., Janowski G., Smusz R., Szumski M. (2021). The influence of chosen plant fillers in PHBV composites on the processing conditions, mechanical properties and quality of molded pieces. Polymers.

[5] Boey J.Y., Lee C.K., Tay G.S. (2022). Factors affecting mechanical properties of reinforced bioplastics: A review. Polymers.

[6] Remila B., Zembouai I., Zaidi L., Kaci M., Kervoëlen A., Bruzaud S. (2025). Surface treatment effects on morphological and property enhancements of poly(3-hydroxybutyrate-co-3-hydroxyvalerate)/Diss fibers (*Ampelodesmos mauritanicus*) biocomposites. Polym. Compos..

[7] Janowski G., Frącz W., Bąk Ł. (2022). The mechanical properties prediction of poly[(3-hydroxybutyrate)-co-(3-hydroxyvalerate)] (PHBV) biocomposites on a chosen example. Materials.

[8] Statistics Poland (2025). Forestry in 2024.

[9] Manterola-Barroso C., Padilla Contreras D., Ondrasek G., Horvatinec J., Gavilán CuiCui G., Meriño-Gergichevich C. (2024). Hazelnut and walnut nutshell features as emerging added-value byproducts of the nut industry: A review. Plants.

[10] Jahanban-Esfahlan A., Jahanban-Esfahlan R., Tabibiazar M., Roufegarinejad L., Amarowicz R. (2020). Recent advances in the use of walnut (*Juglans regia* L.) shell as a valuable plant-based bio-sorbent for the removal of hazardous materials. RSC Adv..

[11] Frącz W., Janowski G., Bąk Ł. (2021). Influence of the alkali treatment of flax and hemp fibers on the properties of PHBV based biocomposites. Polymers.

[12] Mazur K., Kuciel S. (2019). Mechanical and hydrothermal aging behaviour of polyhydroxybutyrate-co-valerate (PHBV) composites reinforced by natural fibres. Molecules.

[13] Du J., Zhao G., Pan M., Zhuang L., Li D., Zhang R. (2017). Crystallization and mechanical properties of reinforced PHBV composites using melt compounding: Effect of CNCs and CNFs. Carbohydr. Polym..

[14] Zheng T., Clemons C.M., Pilla S. (2020). Comparative study of direct compounding, coupling agent-aided and initiator-aided reactive extrusion to prepare cellulose nanocrystal/PHBV (CNC/PHBV) nanocomposite. ACS Sustain. Chem. Eng..

[15] Haham H., Shen M.-Y., Billington S.L., Frank C.W. (2020). Comparison of nanocrystalline cellulose dispersion versus surface nucleation in poly(3-hydroxybutyrate-co-3-hydroxyvalerate) crystallization. SPE Polym..

[16] Doineau E., Rol F., Gontard N., Angellier-Coussy H. (2022). Physical-chemical and structural stability of poly(3HB-co-3HV)/(ligno-)cellulosic fibre-based biocomposites over successive dishwashing cycles. Membranes.

[17] Feijóo P., Marín A., Samaniego-Aguilar K., Sánchez-Safont E., Lagarón J.M., Gámez-Pérez J., Cabedo L. (2023). Effect of the presence of lignin from woodflour on the compostability of PHA-based biocomposites: Disintegration, biodegradation and microbial dynamics. Polymers.

[18] Furgier V., Root A., Heinmaa I., Zamani A., Åkesson D. (2024). Development and characterisation of composites prepared from PHBV compounded with organic waste reinforcements, and their soil biodegradation. Materials.

[19] Mazur K.E., Kuciel S.T., Pieszczek N. (2026). Waste-derived natural fillers in poly(3-hydroxybutyrate-co-3-hydroxyvalerate)-based biocomposites for short-lifespan products: A route toward fully compostable materials. Adv. Sci. Technol. Res. J..

[20] https://shop.helianpolymers.com/products/enmat-y1000p-phbv-sample.

[21] TDS: ENMAT Y1000P. https://api.atomler.com/file/110457086.

[22] (2014). Plastics—Multipurpose Test Specimens.

[23] (2019). Plastics—Methods for Determining the Density of Non-Cellular Plastics.

[24] (2002). Plastics—Multipurpose Test Specimens.

[25] (2010). Plastics—Determination of Tensile Properties.

[26] (2010). Plastics—Determination of Charpy Impact Properties.

[27] (2008). Plastics—Determination of Water Absorption.

[28] Guo Y., Wang L., Chen Y., Luo P., Chen T. (2019). Properties of Luffa Fiber Reinforced PHBV Biodegradable Composites. Polymers.

[29] Samaniego-Aguilar K., Sánchez-Safont E., Rodríguez A., Marín A., Candal M.V., Cabedo L., Gámez-Pérez J. (2023). Valorization of Agricultural Waste Lignocellulosic Fibers for Poly(3-Hydroxybutyrate-Co-Valerate)-Based Composites in Short Shelf-Life Applications. Polymers.

[30] Jin X., Zhang X., Xu C., Nie S. (2019). Effect of bamboo fibers with different coupling agents on the properties of poly(hydroxybutyrate-co-valerate) biocomposites. J. Appl. Polym. Sci..

[31] Janowski G., Frącz W., Bąk Ł., Sikora J.W., Tomczyk A., Mrówka-Nowotnik G., Mossety-Leszczak B. (2025). Effect of Coffee Grounds Content on Properties of PHBV Biocomposites Compared to Similar Composites with Other Fillers. Polymers.

[32] Gómez-Gast N., López-Cuellar M.R., Vergara-Porras B., Vieyra H. (2022). Biopackaging Potential Alternatives: Bioplastic Composites of Polyhydroxyalkanoates and Vegetal Fibers. Polymers.

[33] Raza Z.A., Noor S., Khalil S. (2019). Recent developments in the synthesis of poly(hydroxybutyrate) based biocomposites. Biotechnol. Prog..

[34] Meléndez-Rodríguez B., Torres-Giner S., Aldureid A., Cabedo L., Lagarón J.M. (2019). Reactive Melt Mixing of Poly(3-Hydroxybutyrate)/Rice Husk Flour Composites with Purified Biosustainably Produced Poly(3-Hydroxybutyrate-co-3-Hydroxyvalerate). Materials.

[35] Righetti M.C., Cinelli P., Mallegni N., Stäbler A., Lazzeri A. (2019). Thermal and Mechanical Properties of Biocomposites Made of Poly(3-hydroxybutyrate-co-3-hydroxyvalerate) and Potato Pulp Powder. Polymers.

[36] Bhardwaj R., Mohanty A.K., Drzal L.T., Pourboghrat F., Misra M. (2006). Renewable Resource-Based Green Composites from Recycled Cellulose Fiber and Poly(3-hydroxybutyrate-co-3-hydroxyvalerate) Bioplastic. Biomacromolecules.

[37] Muthuraj R., Misra M., Mohanty A.K. (2017). Reactive compatibilization and performance evaluation of miscanthus biofiber reinforced poly(hydroxybutyrate-co-hydroxyvalerate) biocomposites. J. Appl. Polym. Sci..

[38] Mathel V., Pennells J., Vandi L.-J., Martin D. (2026). Sustainable manufacture of poly(3-hydroxybutyrate-co-3-hydroxyvalerate)-based biocomposites incorporating sorghum-derived microfibrillated cellulose. Ind. Crops Prod..

[39] Santos F., Vargas P., Massano A., Carvalho B., Fonseca A.C., Mitchell G., Santos B., Mateus A., Encarnação T. (2026). Biocomposite of poly(3-hydroxybutyrate-co-3-hydroxyvalerate) (PHBV), wood fibres and additives: Physicochemical properties and processability through different techniques. Eur. J. Wood Prod..

[40] Yang H., Yan R., Chen H., Lee D.H., Zheng C. (2007). Characteristics of hemicellulose, cellulose and lignin pyrolysis. Fuel.

[41] David G., Gontard N., Angellier-Coussy H. (2019). Mitigating the Impact of Cellulose Particles on the Performance of Biopolyester-Based Composites by Gas-Phase Esterification. Polymers.

[42] Azwa Z.N., Yousif B.F., Manalo A.C., Karunasena W. (2013). A review on the degradability of polymeric composites based on natural fibres. Mater. Des..

[43] Kord B. (2013). Assessment of long-term water absorption in natural fiber reinforced thermoplastic composites influenced by filler rate and compatibilizer treatment. J. Thermoplast. Compos. Mater..

[44] Zhao X., Presnell K., Tolu L., Vodovotz Y. (2024). Value-added use of spent coffee ground for low-cost poly(3-hydroxybutyrate-co-3-hydroxyvalerate) biocomposite development. J. Appl. Polym. Sci..

[45] Janowski G., Wójcik M., Krešić I., Frącz W., Bąk Ł., Gajdoš I., Spišák E. (2026). The Influence of Sunflower Seed Hull Content on the Mechanical, Thermal, and Functional Properties of PHBV-Based Biocomposites. Materials.

[46] Iggui K., Kaci M., Le Moigne N., Bergeret A. (2021). Effects of hygrothermal aging on chemical, physical, and mechanical properties of poly(3-hydroxybutyrate-co-3-hydroxyvalerate)/Cloisite 30B bionanocomposite. Polym. Compos..

[47] Laycock B., Nikolić M., Colwell J.M., Gauthier E., Halley P.J., Bottle S.E., George G.A. (2017). Lifetime prediction of biodegradable polymers. Prog. Polym. Sci..

[48] Liu H., Pancholi M., Stubbs J.T., Raghavan D. (2010). Influence of hydroxyvalerate composition of polyhydroxy butyrate valerate (PHBV) copolymer on bone cell viability and in vitro degradation. J. Appl. Polym. Sci..

[49] Sultana N., Khan T.H. (2012). In Vitro Degradation of PHBV Scaffolds and nHA/PHBV Composite Scaffolds Containing Hydroxyapatite Nanoparticles for Bone Tissue Engineering. J. Nanomater..

[50] Bonartsev A.P., Boskhomdzhiev A., Voinova V.V., Makhina T.A., Myshkina V.L., Yakovlev S., Zharkova I.I., Filatova E.V., Zernov A.L., Bagrov D. (2012). Degradation of Poly(3-hydroxybutyrate) and its Derivatives: Characterization and Kinetic Behavior. Chem. Chem. Technol..

[51] Abdallah Y., Ben Cheikh R. (2026). Manufacturing, characterization and accelerated weathering of biocomposite made of poly (β-hydroxybutyrate)-co-poly(β-hydroxyvalerate) and non-woven alfa fibers. Compos. Adv. Mater..

