# Supplementary material for: Structure–Property–Degradation Relationships in PHBV Biocomposites Filled with Cellulose, Walnut Shell, and Wood Fibres
Source: Materials (Basel). 2026 Oct 31;19(21):4638. doi: 10.3390/ma19214638 (PMC13764985; doi:10.3390/ma19214638)
Supplement: Supplementary file 1 [file materials-19-04638-s001.zip › materials-4619136-supplementary.pdf]

Supplementary Material

ANOVA of PHBV composite properties

Analysis assumptions and methods

A sample size of n = 5 was taken for each group and each parameter analyzed. Seven formulations were compared: PHBV, 10C, 20C, 10R, 20R, 10W, and 20W, with N = 35 per parameter. Here, C represents cellulose, R denotes walnut shells, and W indicates wood fibres; 10 and 20 refer to the filler content in weight percent. The input data comprised the means and standard deviations as reported in Table 2 (density), Table 5 (contact angles and surface energy), and Table 6 (mechanical properties at 23 °C). Values following ± were considered as standard deviations. It was assumed that observations were independent and that there was approximate normality within groups. Welch's one-way ANOVA was employed to address unequal variances. The factor under consideration was material formulation, and the null hypothesis posited the equality of the seven group means. The significance level was set at $\alpha = 0.05$. Holm's correction was applied across the 10 ANOVA tests, and Games–Howell post hoc comparisons were conducted for all 21 pairs within each parameter.

ANOVA results

Table S1. Welch's ANOVA; df1 = 6. Significance is based on Holm-adjusted p values.

Parameter	Welch F	df2	p	Holm p	Significant
Tensile strength (MPa)	415.82	11.30	1.42×10^{-12}	8.51×10^{-12}	yes
Young's modulus (MPa)	36.09	12.24	4.34×10^{-7}	8.67×10^{-7}	yes
Elongation at break (%)	6.22	12.26	0.0034	0.0034	yes
Charpy impact strength (kJ/m²)	242.30	12.15	6.43×10^{-12}	3.21×10^{-11}	yes
Density (g/cm³)	251.87	11.99	6.72×10^{-12}	3.21×10^{-11}	yes
Water contact angle (°)	4515.26	12.32	7.46×10^{-20}	6.72×10^{-19}	yes
Diiodomethane contact angle (°)	6089.56	12.34	1.13×10^{-20}	1.13×10^{-19}	yes
Dispersive component γd (mJ/m²)	225.38	12.16	9.61×10^{-12}	3.21×10^{-11}	yes
Polar component γp (mJ/m²)	435.58	12.34	1.25×10^{-13}	8.73×10^{-13}	yes
Surface free energy γ (mJ/m²)	499.25	12.31	5.83×10^{-14}	4.66×10^{-13}	yes

Statistically significant differences among formulations were found for all 10 parameters (Holm-adjusted $p < 0.005$). Differences relative to PHBV are presented in Tables S2 and S3.

Comparisons with PHBV

Each cell gives the mean difference Δ = composite − PHBV and the Games–Howell p value. A + sign indicates an increase and a − sign a decrease relative to PHBV. Values of p < 0.05 are shown in bold. The Games–Howell adjustment covers 21 pairs separately for each parameter.

Table S2. Mechanical properties at 23 °C and density.

Material	Tensile strength (MPa)	Young's modulus (MPa)	Elongation (p.p.)	Impact strength (kJ/m²)	Density (g/cm³)
10C	−2.1<br>p = 0.4790	−104<br>p = 0.4134	+0.3<br>p = 0.3170	+4.2<br>p < 0.001	+0.046<br>p < 0.001
20C	−5.5<br>p = 0.0337	+228<br>p = 0.0076	0.0<br>p = 1.0000	+0.8<br>p < 0.001	+0.048<br>p < 0.001
10R	+0.6<br>p = 0.9956	+287<br>p = 0.2654	+0.2<br>p = 0.6972	+1.7<br>p = 0.0034	+0.009<br>p < 0.001
20R	−6.3<br>p = 0.0189	+175<br>p = 0.0565	0.0<br>p = 1.0000	+0.4<br>p = 0.0612	+0.032<br>p < 0.001
10W	+2.0<br>p = 0.5107	+1101<br>p < 0.001	0.0<br>p = 1.0000	+0.6<br>p = 0.0096	+0.007<br>p = 0.2024
20W	−5.9<br>p = 0.0165	+1713<br>p = 0.0089	−0.5<br>p = 0.0420	−0.6<br>p = 0.2985	+0.026<br>p < 0.001

Table S3. Contact angles and surface energy.

Material	Water contact angle (°)	Diiodomethane contact angle (°)	γd (mJ/m²)	γp (mJ/m²)	γ (mJ/m²)
10C	−7.50<br>p < 0.001	+10.98<br>p < 0.001	−4.34<br>p < 0.001	+3.78<br>p < 0.001	−0.56<br>p = 0.6687
20C	−13.47<br>p < 0.001	+0.82<br>p < 0.001	−0.28<br>p = 0.4591	+5.38<br>p < 0.001	+5.10<br>p < 0.001
10R	−4.96<br>p < 0.001	+3.89<br>p < 0.001	−1.39<br>p = 0.0033	+2.01<br>p < 0.001	+0.62<br>p = 0.3319
20R	−13.23<br>p < 0.001	−2.23<br>p < 0.001	+0.70<br>p = 0.0108	+5.00<br>p < 0.001	+5.70<br>p < 0.001
10W	−12.51<br>p < 0.001	+7.20<br>p < 0.001	−2.70<br>p < 0.001	+5.62<br>p < 0.001	+2.92<br>p < 0.001
20W	−18.27<br>p < 0.001	−3.79<br>p < 0.001	+1.16<br>p < 0.001	+7.26<br>p < 0.001	+8.42<br>p < 0.001

γd — dispersive component; γp — polar component; γ — total surface free energy; p.p. — percentage points.
